# Supplementary figures and images for: Germline mutation rates and fine-scale recombination parameters in zebra finch
Source: PLoS Genet. 2025 Apr 15;21(4):e1011661. doi: 10.1371/journal.pgen.1011661 (PMC12047795; doi:10.1371/journal.pgen.1011661)

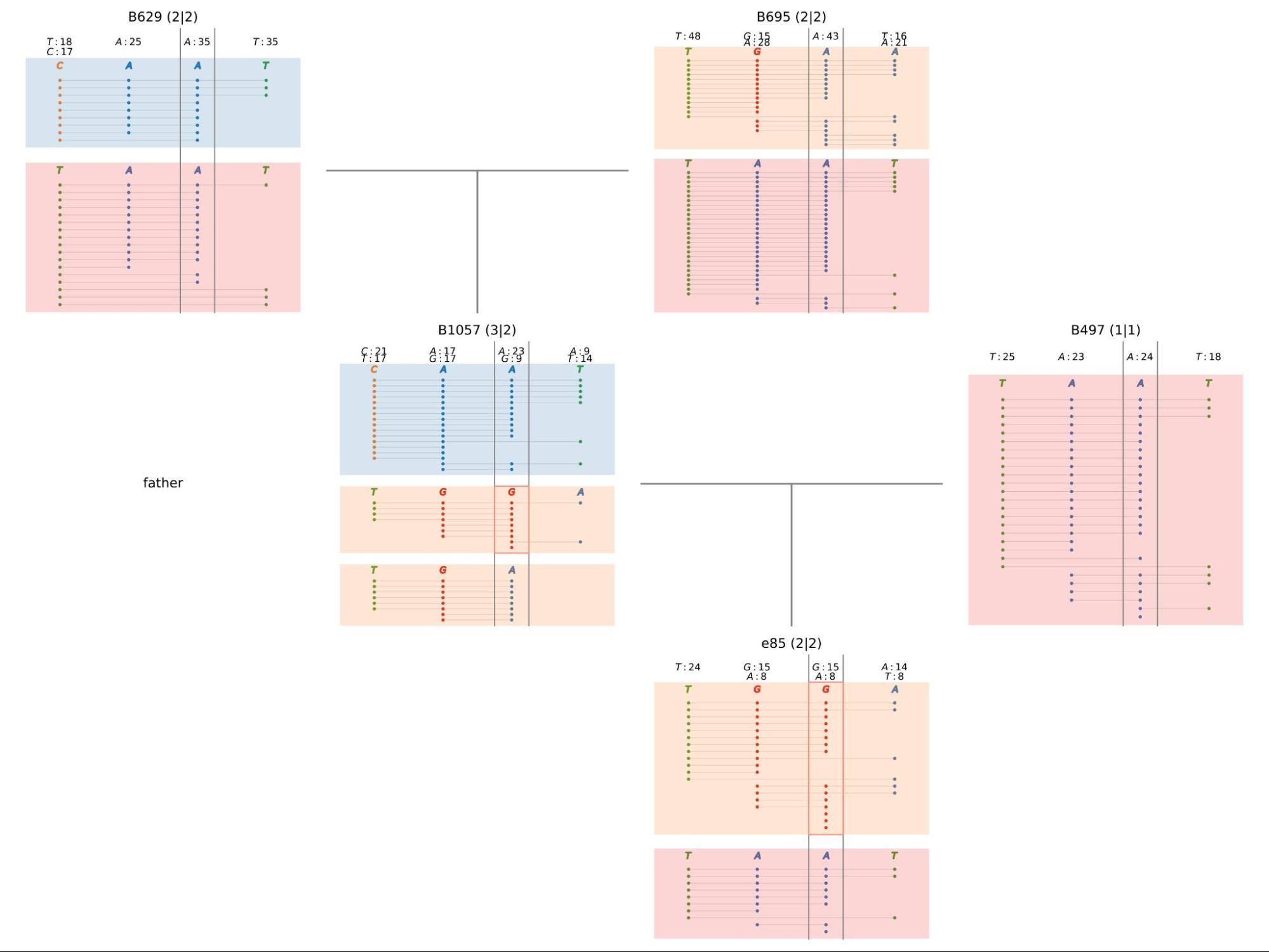

Supplement: S1 Fig — For each of the five individuals in this pedigree, the nucleotides (circles) carried by pairs of sequencing reads spanning multiple informative heterozygous sites (horizontal lines) are shown. The mutated position is indicated by gray vertical lines, and colors represent groups of reads supporting the same haplotype (excluding the mutated position). In haplotypes carrying the DNM, the mutated position is highlighted by a red rectangle. In carrier individuals, all sequencing reads are represented for the mutated position (regardless of whether they span multiple informative sites). In the first generation (parents of the proband), the mother is positioned on the left. The annotation left to the panel of the proband shows the parental haplotype carrying the DNM. Each panel indicates the number of reads supporting each allele (top side), regardless of whether they span more than one informative site. (TIF) [file pgen.1011661.s001.tif]

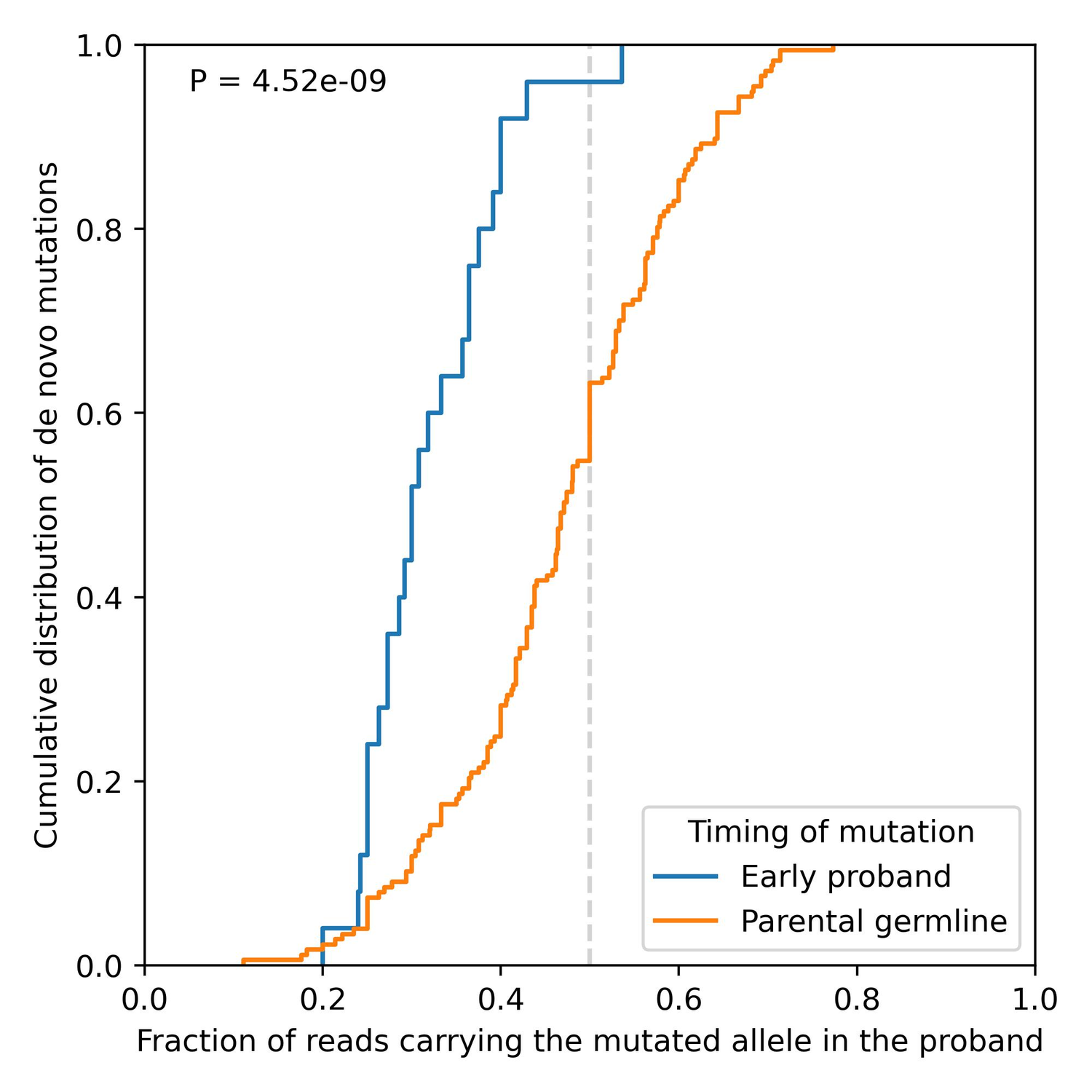

Supplement: S2 Fig — Cumulative distribution of the fraction of sequencing reads in the proband carrying the mutated allele, for DNMs inferred to have occurred in either parental germlines (orange) and in the early development of the proband (blue). The expected fraction of ½ for inherited variants is shown as a vertical dashed line. The p-value is obtained from a Kolmogorov-Smirnov test. (TIF) [file pgen.1011661.s002.tif]

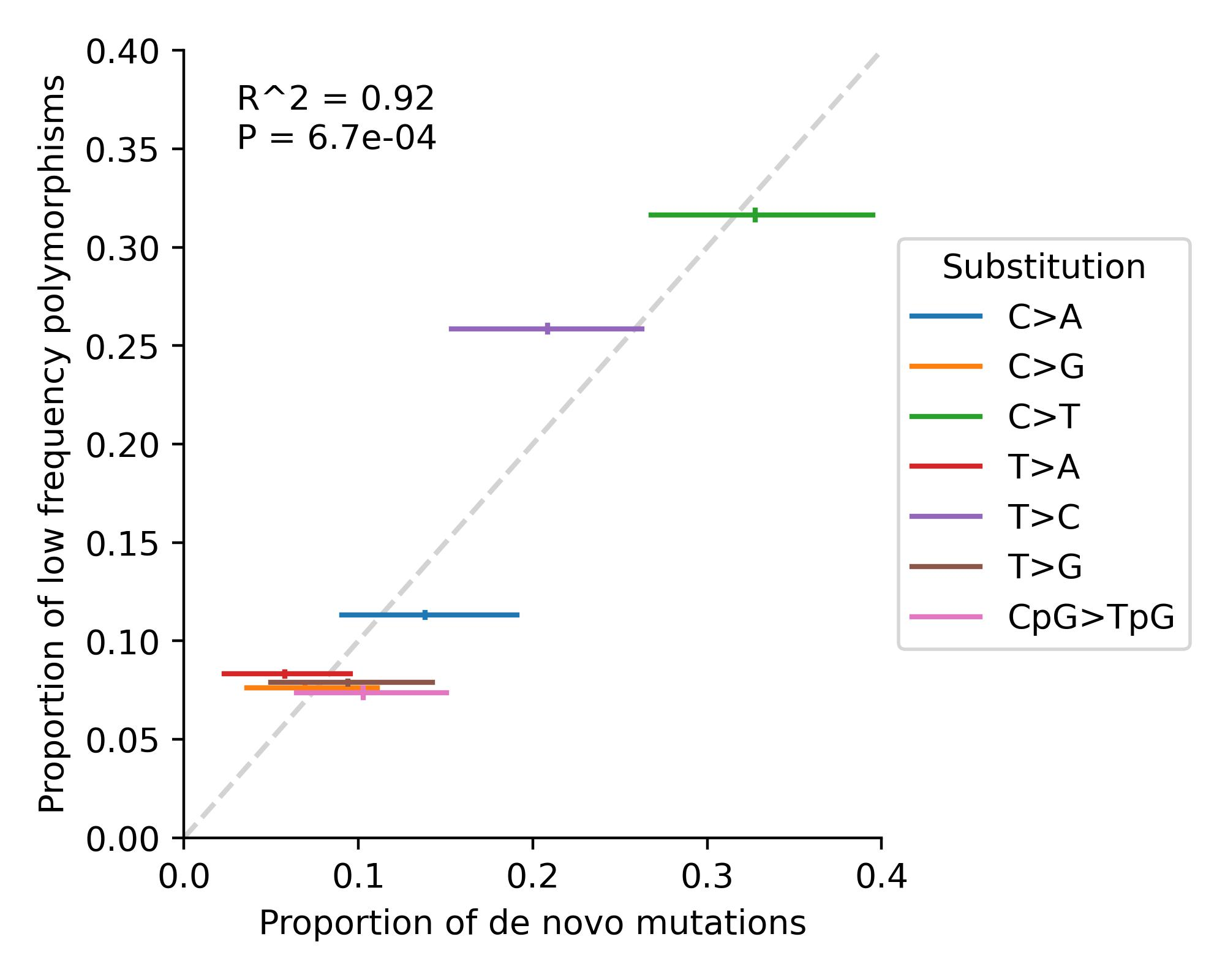

Supplement: S3 Fig — Proportion of each substitution type in DNMs inferred from pedigree sequencing (x-axis) versus in low-frequency polymorphisms segregating in a dataset of 27 non-closely related individuals (y-axis). 95% CIs (lines) were obtained by bootstrapping 5 Mb autosomal windows 500 times. (TIF) [file pgen.1011661.s003.tif]

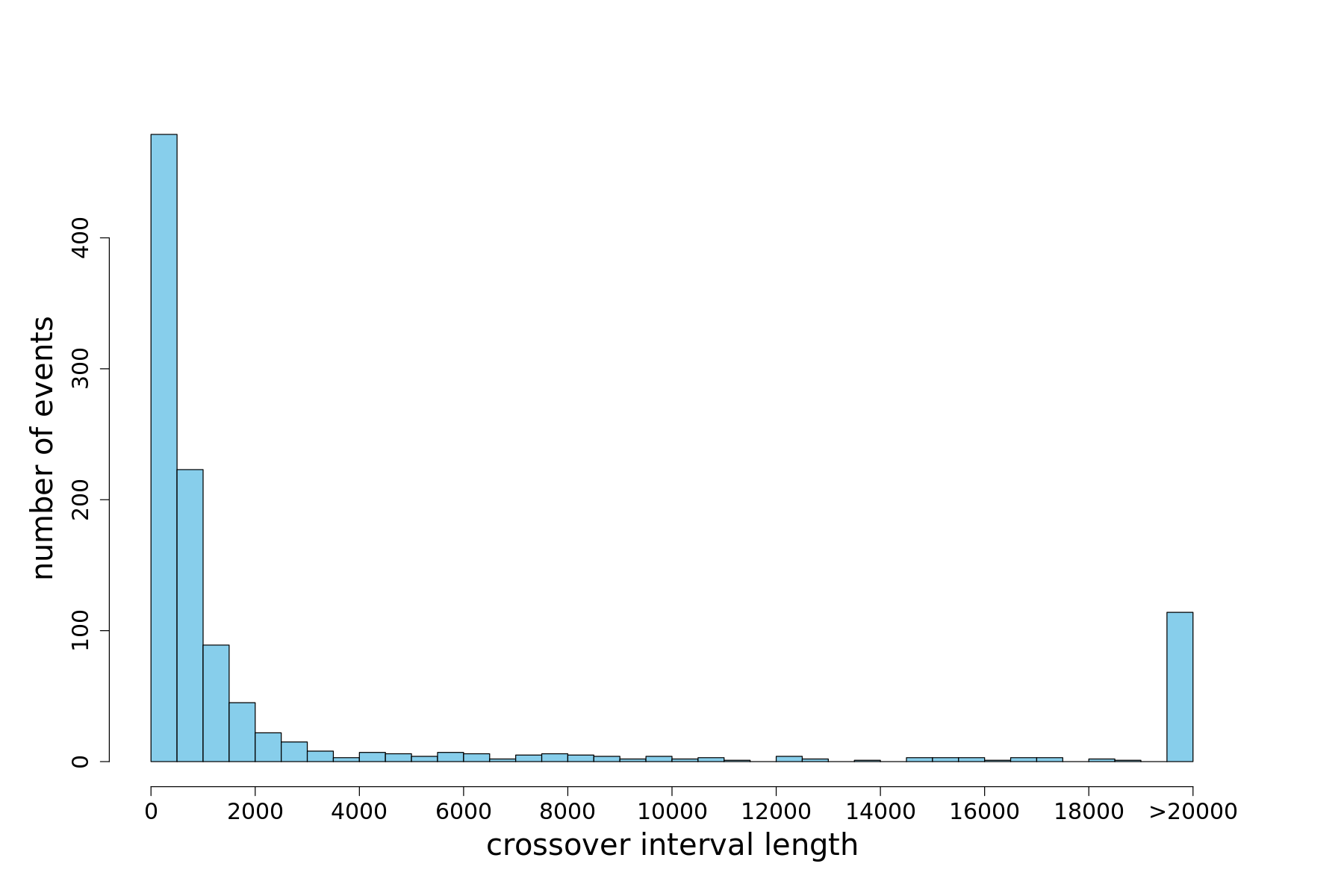

Supplement: S4 Fig — Intervals larger than 20,000 bps are shown in one bin. (TIF) [file pgen.1011661.s004.tif]

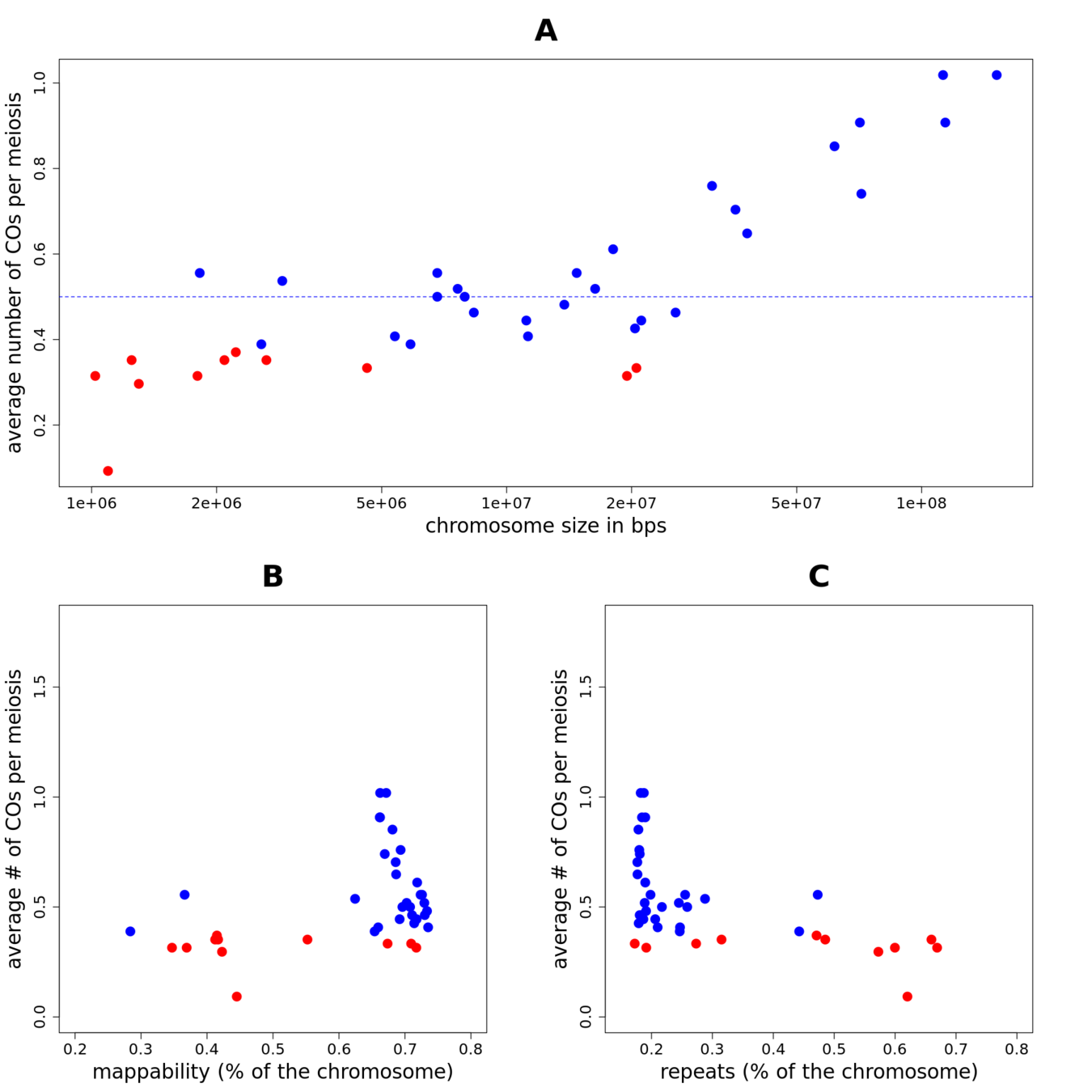

Supplement: S5 Fig — (A) Average number of crossovers observed per meiosis for the 39 autosomes. The X axis is on a log-scale. The blue dashed line represents the minimum number of crossovers expected per chromosome per meiosis. (B) Average number of crossovers observed per meiosis as a function of the mappability of the chromosome. (C) Average number of crossovers observed per meiosis as a function of the proportion of repeat elements in the chromosome. For each panel, the red dots indicate the chromosomes for which the average number is significantly smaller than 0.5 (p < 0.05, using an exact binomial test). (TIF) [file pgen.1011661.s005.tif]

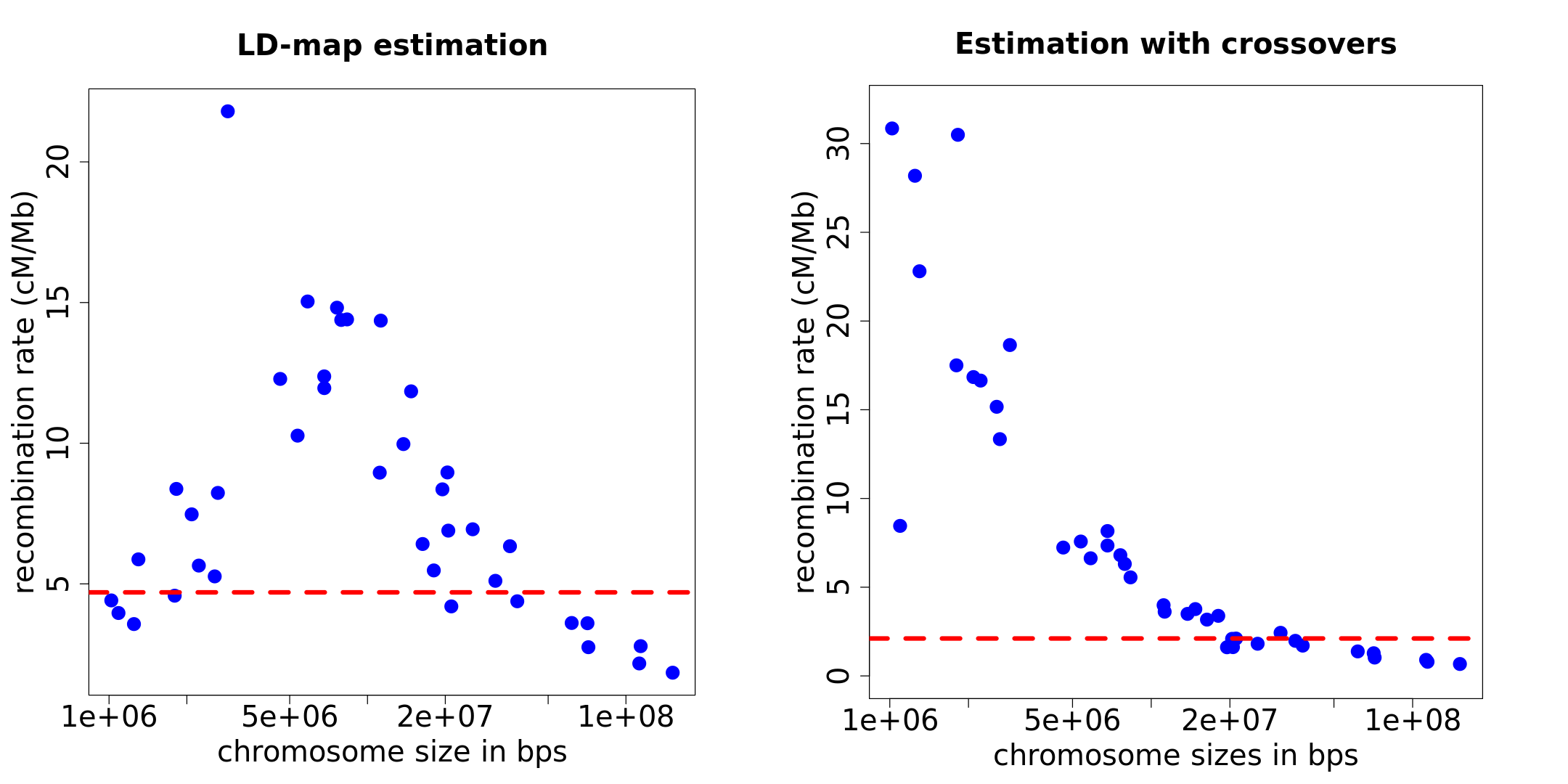

Supplement: S6 Fig — The left panel shows estimates from the LD-based map; the dashed red line represents the mean recombination rate for the whole genome, including micro-chromosomes (4.68 cM/MB). The right panel shows estimates from crossovers; the red dashed line is the mean recombination rate for the whole genome is 2.28 cM/Mb. The comparison of these two plots suggests that estimates of recombination rates for chromosomes <40 Mb are unreliable, consistent with simulations [51]. (TIF) [file pgen.1011661.s006.tif]

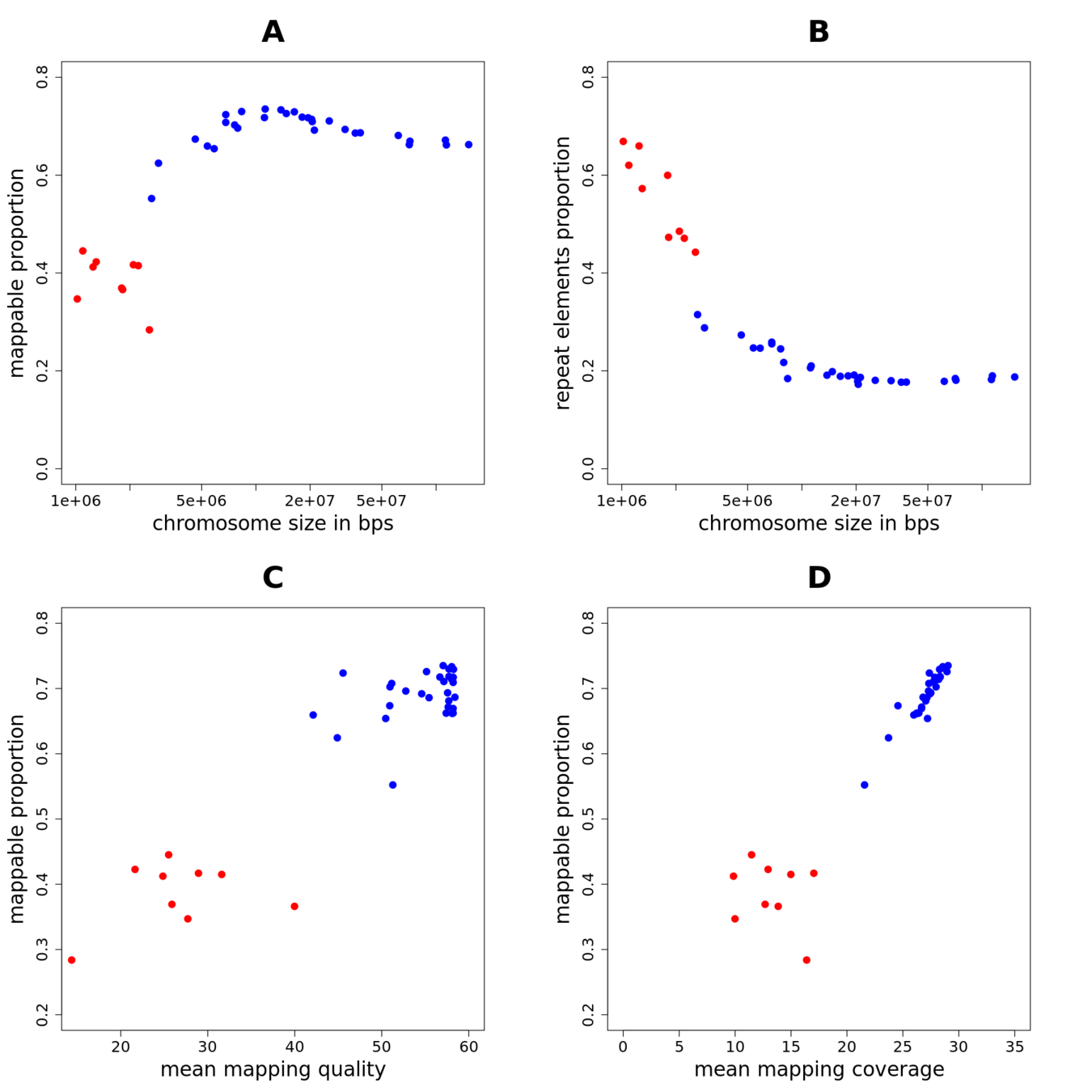

Supplement: S7 Fig — (A) Fraction of the chromosome that is mappable (see Methods) as a function of chromosome size. (B) Proportion of repeat elements per chromosome as a function of chromosome size. The proportion of repeat elements was estimated by counting the proportion of positions, for each chromosome, that is assigned as a repeat in the softmask version of the genome. (C) Proportion of the chromosome that is mappable as a function of the mean mapping quality for each chromosome. (D) Proportion of the chromosome that is mappable as a function of the mean mapping coverage for each chromosome. For each panel, the red dots are the chromosomes that have been removed from the NCO analysis. The blue dots are the chromosomes that meet our mapping quality and mapping coverage threshold (see Methods). (TIF) [file pgen.1011661.s007.tif]

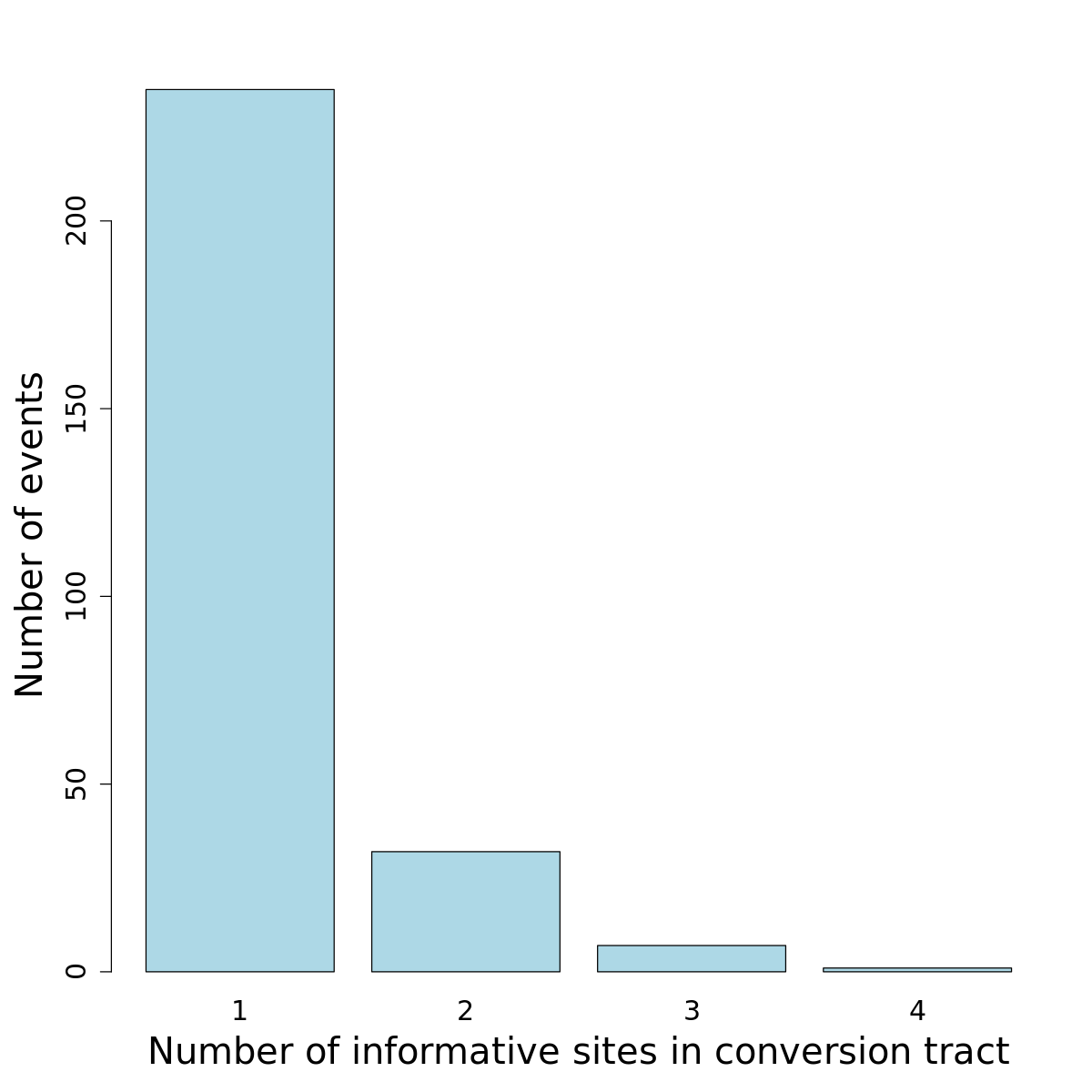

Supplement: S8 Fig — (TIF) [file pgen.1011661.s008.tif]

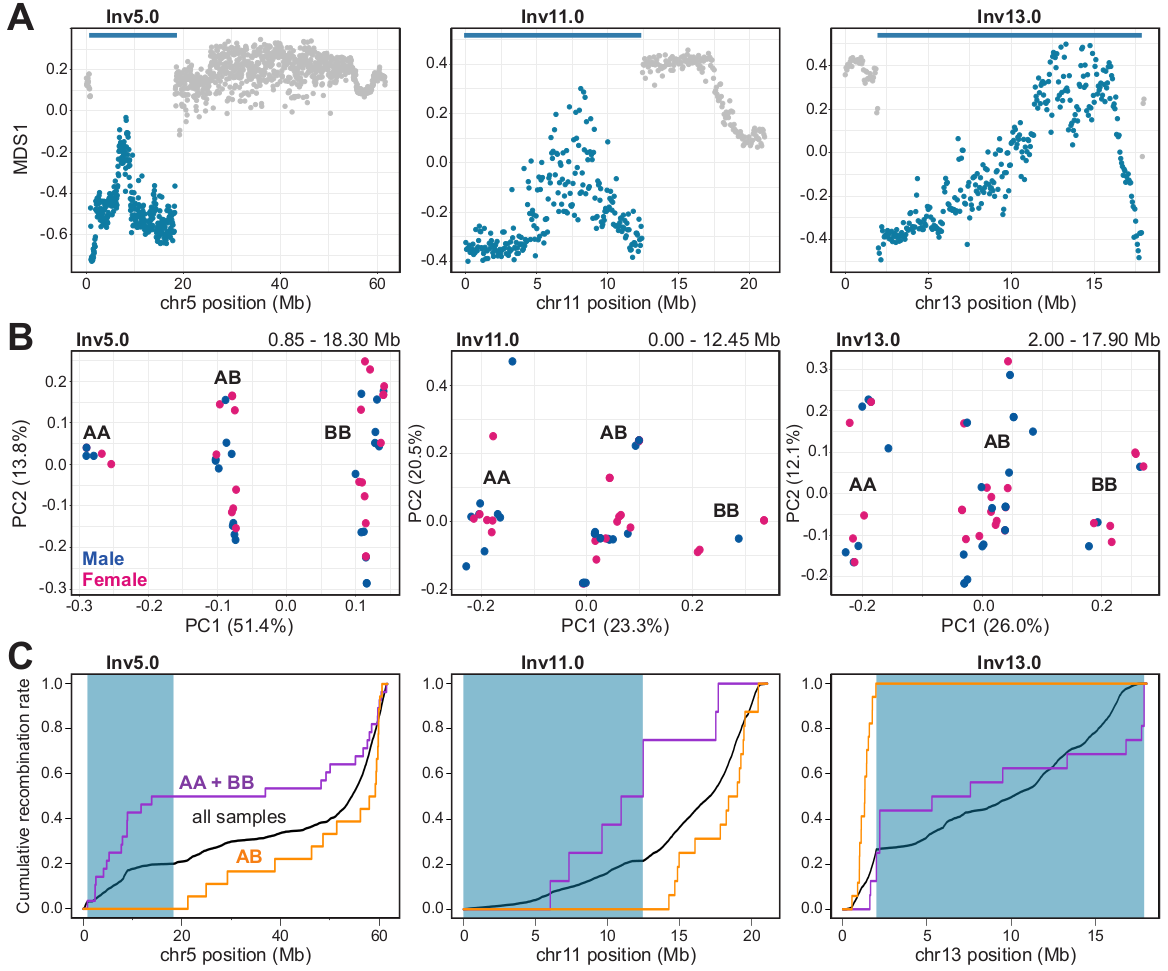

Supplement: S10 Fig — (A) Local PCA for three autosomal chromosomes harboring inversion polymorphisms in the zebra finch, with each point representing a 50 kb window. The distance between the relatedness structure of individual windows was evaluated using multidimensional scaling (MDS) and is represented using the MDS1 axis. Windows colored in blue belong to outlier regions associated with the candidate inversions, with the length of each inversion represented as a horizontal blue bar at the top. (B) Consistent with the occurrence of inversion polymorphisms, PCA using variants from the entirety of each outlier region recover three distinct clusters of samples along PC1. These clusters correspond to either group of homokaryotypic (i.e., groups AA and BB) individuals at opposite ends of PC1 and a group of heterokaryotypic individuals (i.e., group AB) immediately in between. Homokaryotype group assignment (i.e., AA vs. BB) was done arbitrarily for each inversion from left to right along PC1 and is not necessarily consistent with labeling in [83]. The name of each inversion is given at the top left and the approximate coordinates of each inversion is at the top right of each panel, respectively. Points are color-coded by sex for male (blue) or female (pink). (C) Cumulative distributions of crossovers (purple and orange) and population recombination rate inferred from LD-map (black) for each autosome harboring an inversion polymorphism. Crossovers from homokaryotypic and heterokaryotypic individuals represented in purple and orange, respectively. The location of each inversion is shown in blue. (TIF) [file pgen.1011661.s010.tif]

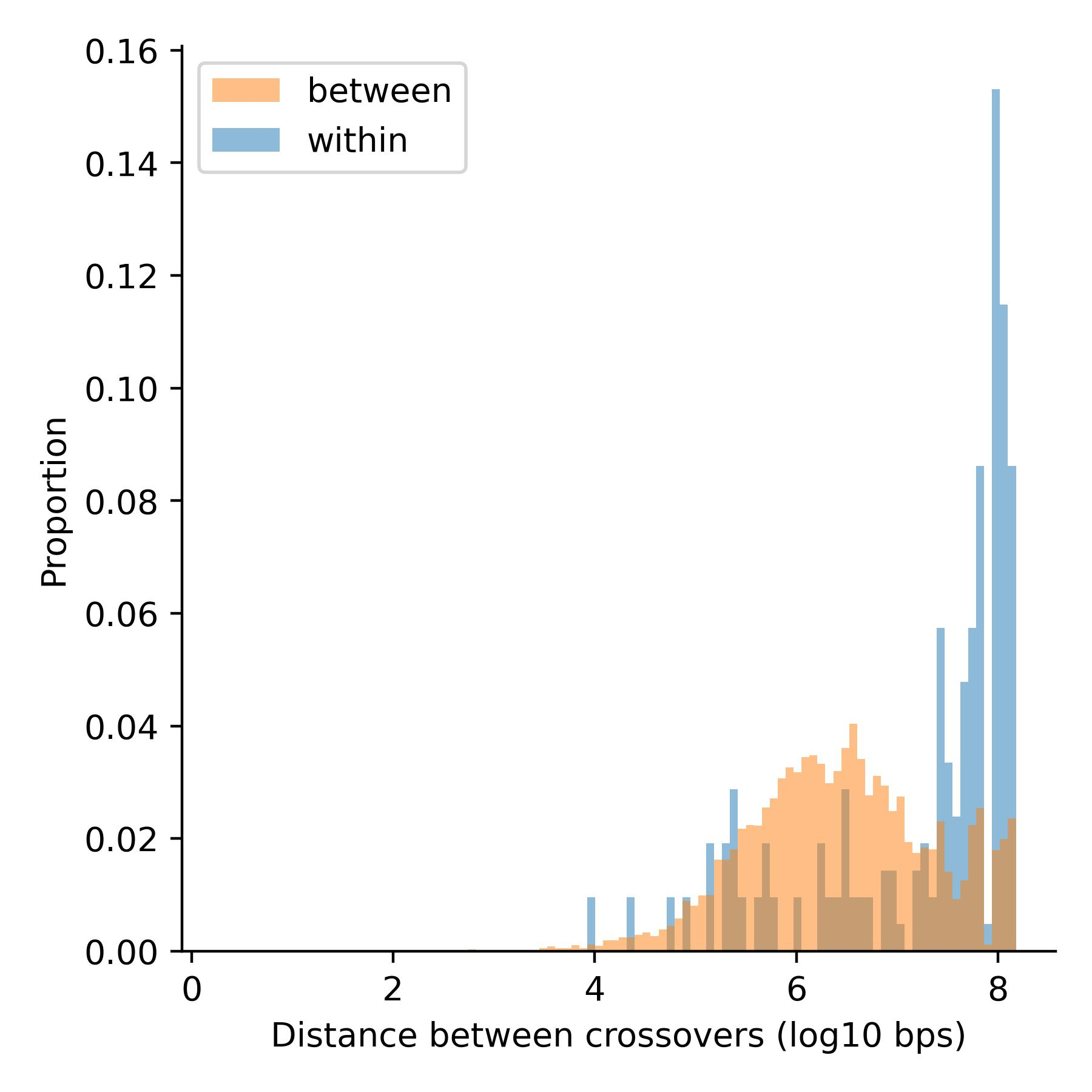

Supplement: S11 Fig — Comparisons of events that occurred within the same meiosis (“within”, blue) or a different meiosis (“between”, orange). (TIF) [file pgen.1011661.s011.tif]

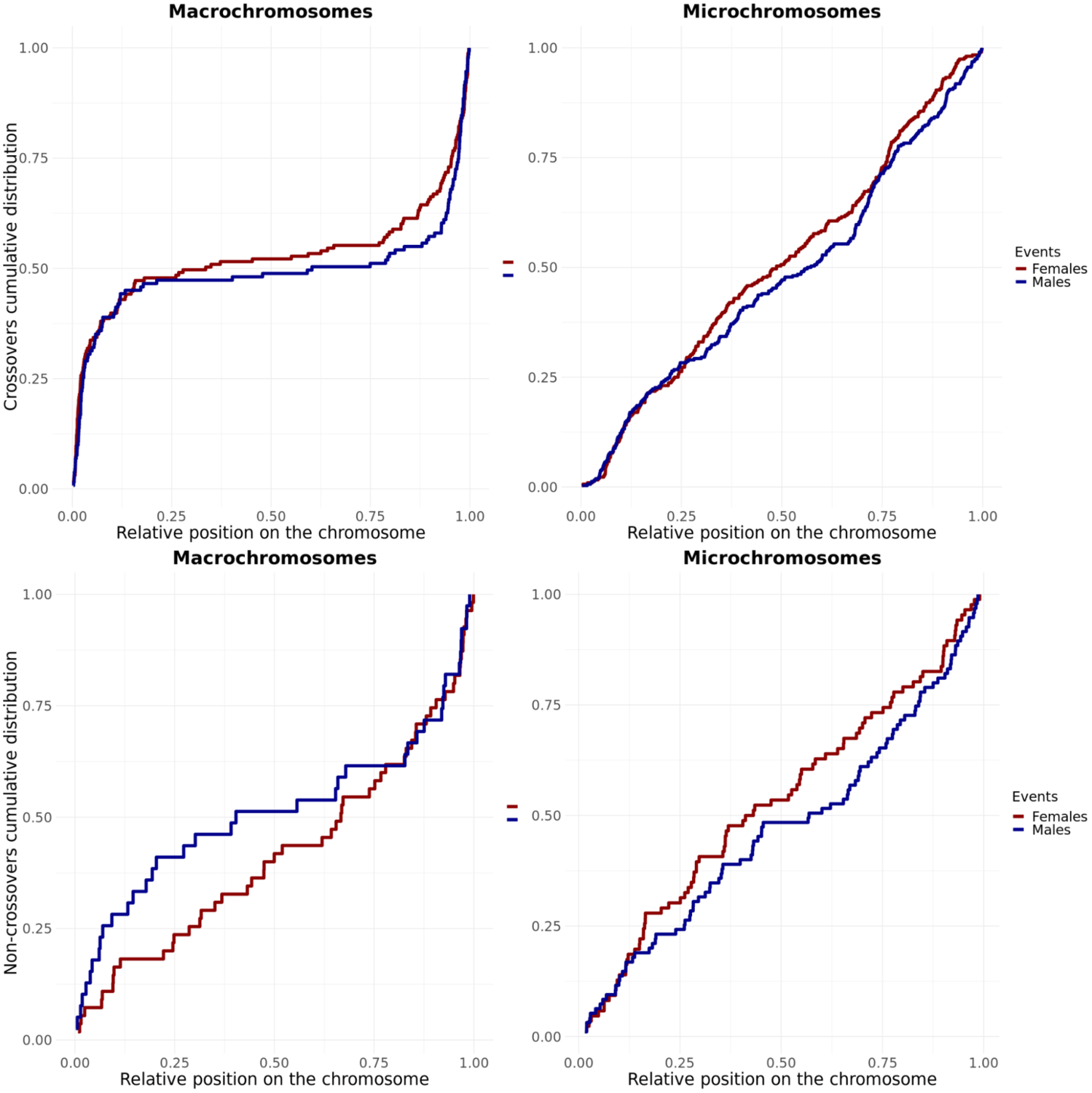

Supplement: S12 Fig — Top panel: Cumulative distribution of crossover events (females in red and males in blue), for macro-chromosomes (left) and micro-chromosomes (right). The position of the events is normalized by the size of the chromosomes. For crossovers, the p-values for Kolmogorov-Smirnov tests comparing the two sexes are 0.31 and 0.34 for the macro-chromosomes and the micro-chromosomes, respectively. Bottom panel: Cumulative distribution of non-crossover events (females in red and males in blue), for macro-chromosomes (left) and micro-chromosomes (right). The position of events is normalized by the size of the chromosomes. For non-crossovers, the p-values for Kolmogorov-Smirnov tests comparing the two sexes are 0.15 and 0.32 for the macro-chromosomes and the micro-chromosomes, respectively. For a comparison of the sex-averaged distribution on macro- vs micro-chromosomes, see S4 Table. (TIF) [file pgen.1011661.s012.tif]

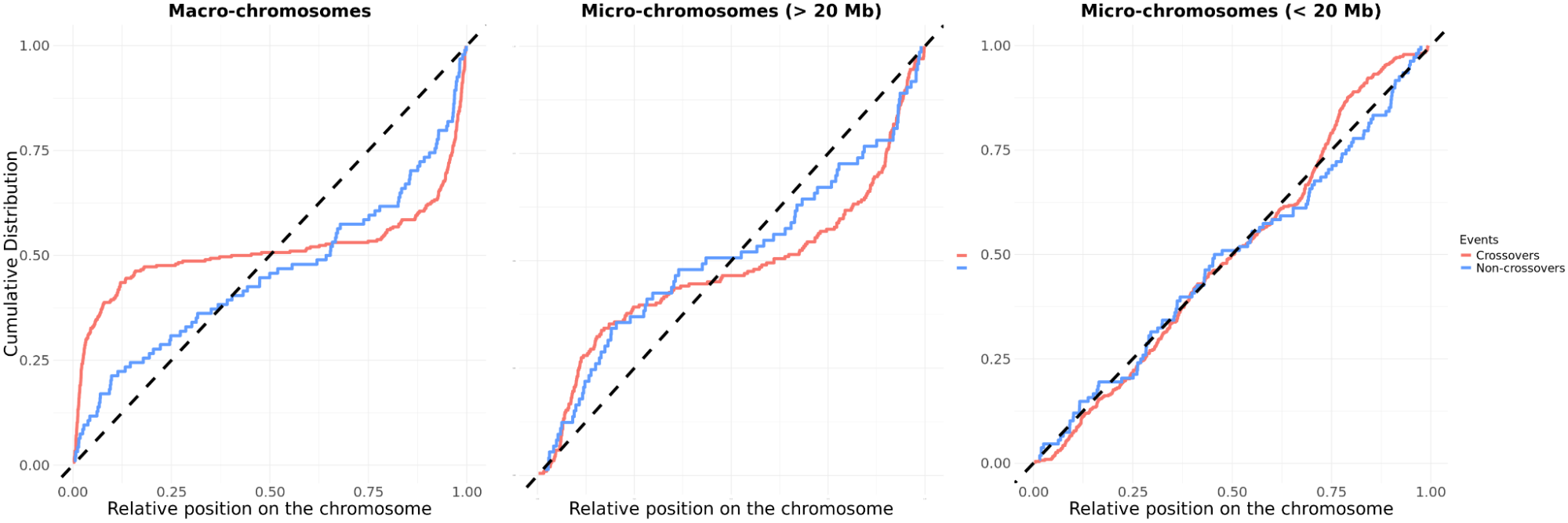

Supplement: S13 Fig — Same as in Fig 2, but considering chromosomes shorter than 20 Mb separately from those between 20 and 40 Mb and macro-chromosomes. (TIF) [file pgen.1011661.s013.tif]

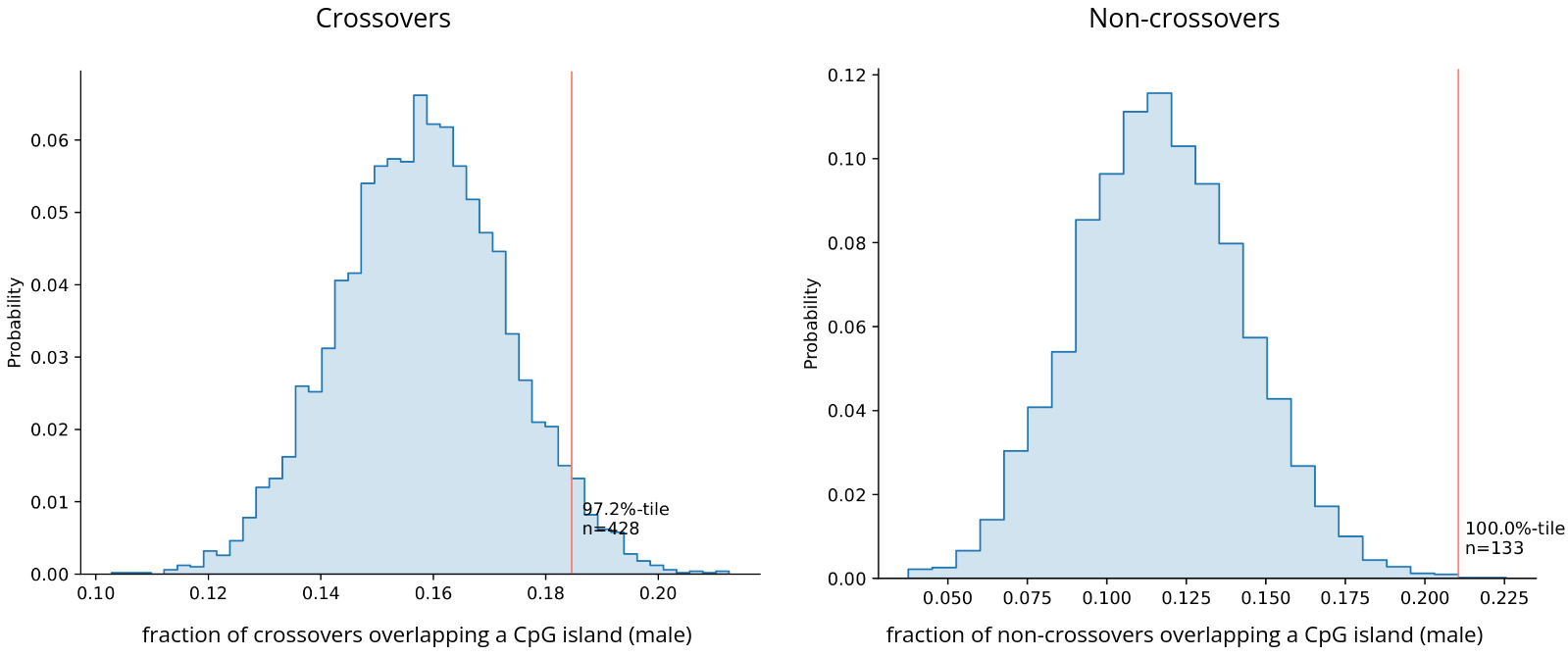

Supplement: S14 Fig — Fractions of crossovers (left) and non-crossovers (right) identified in males meioses within 100 bps of a CpG island. The vertical lines show the observed overlap. The distribution for the overlap expected by chance is shown as a histogram, obtained by randomly shuffling all the events within a 2.5 Mb window on each side of their original location, matching for the GC content and ensuring similar mappability (see Methods for details). (TIF) [file pgen.1011661.s014.tif]

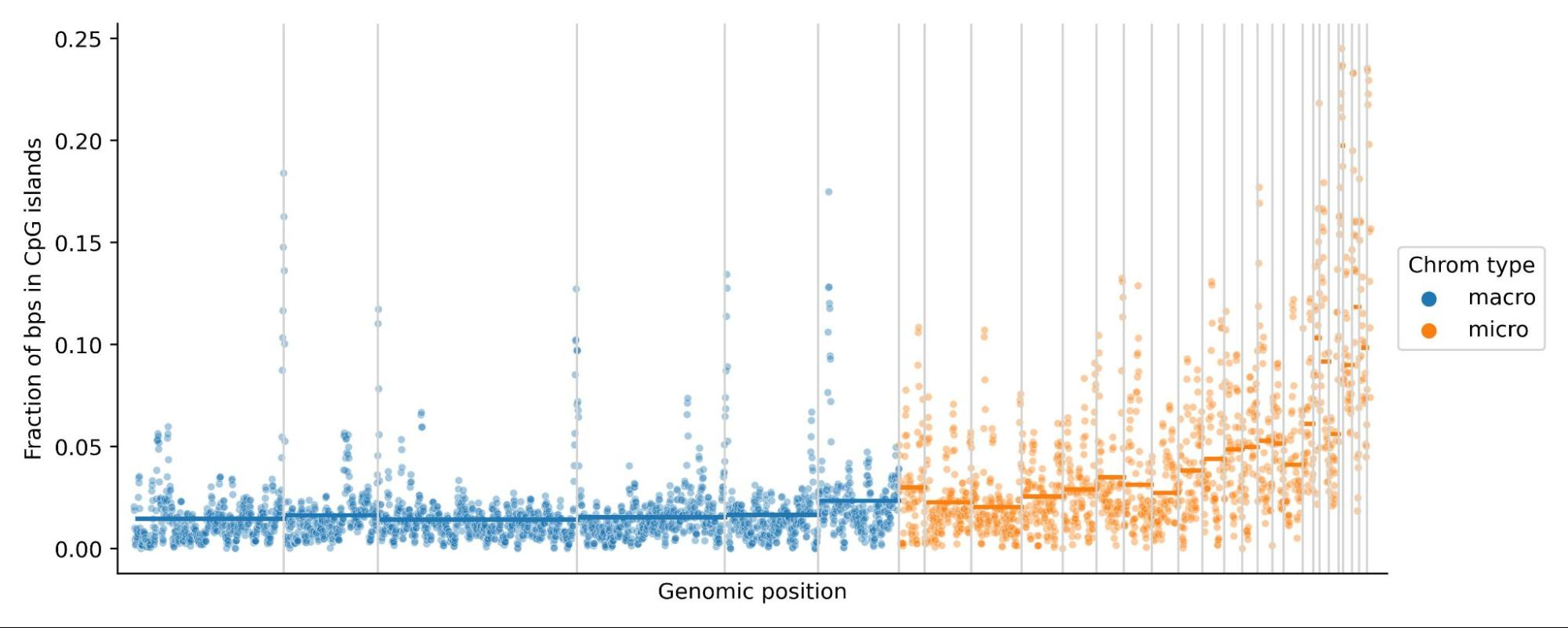

Supplement: S15 Fig — The fraction of bps within CpG islands in consecutive genomic windows of 1 Mb, for the 30 autosomes for which we identified recombination events (see Methods). Vertical gray indicates transitions between different chromosomes. Horizontal lines show the mean CpG island density for the corresponding chromosome. (TIF) [file pgen.1011661.s015.tif]

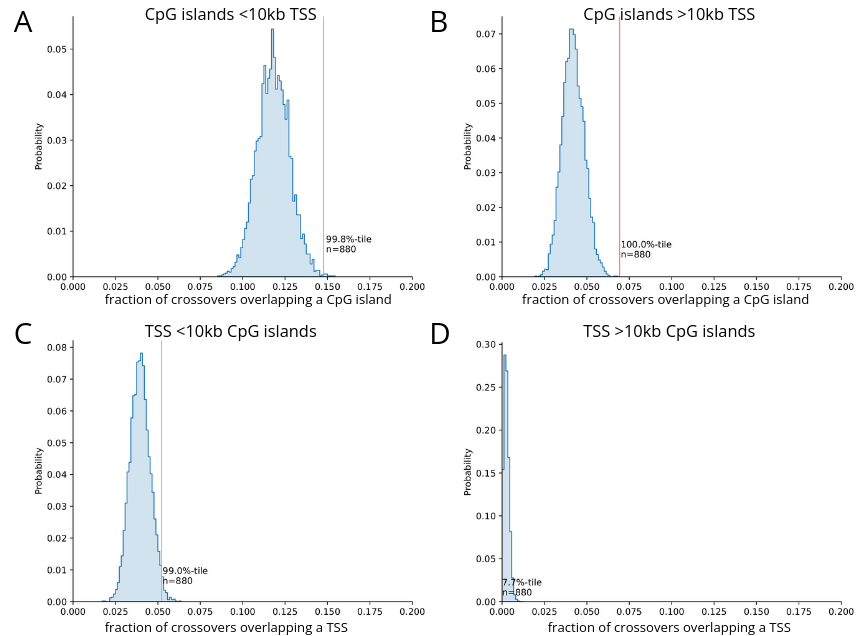

Supplement: S16 Fig — Fraction of crossover events overlapping (A) a CpG island at most 10 kb away from a TSS, (B) a CpG island farther than 10 kb from a TSS, (C) a TSS at most 10 kb away from a CpG island, and (D) a TSS farther than 10 kb from a CpG island. To account for the difference in width between CpG islands and TSSs, we considered that a crossover event overlaps a CpG island (A and B) and a TSS (C and D) if it is closer than 100 bps or 500 bps, respectively. The observed overlap is shown by red vertical lines. The overlap expected by chance is shown as a histogram; it was obtained by randomly shuffling all crossovers events 5,000 times within 2.5 Mb of their original location, ensuring the shuffled location had a similar GC content and mappability (see Methods). TSSs were identified from the genome annotation (GCF_003957565.2) by keeping the positions annotated as “start_codon” (n = 20,400). (TIF) [file pgen.1011661.s016.tif]

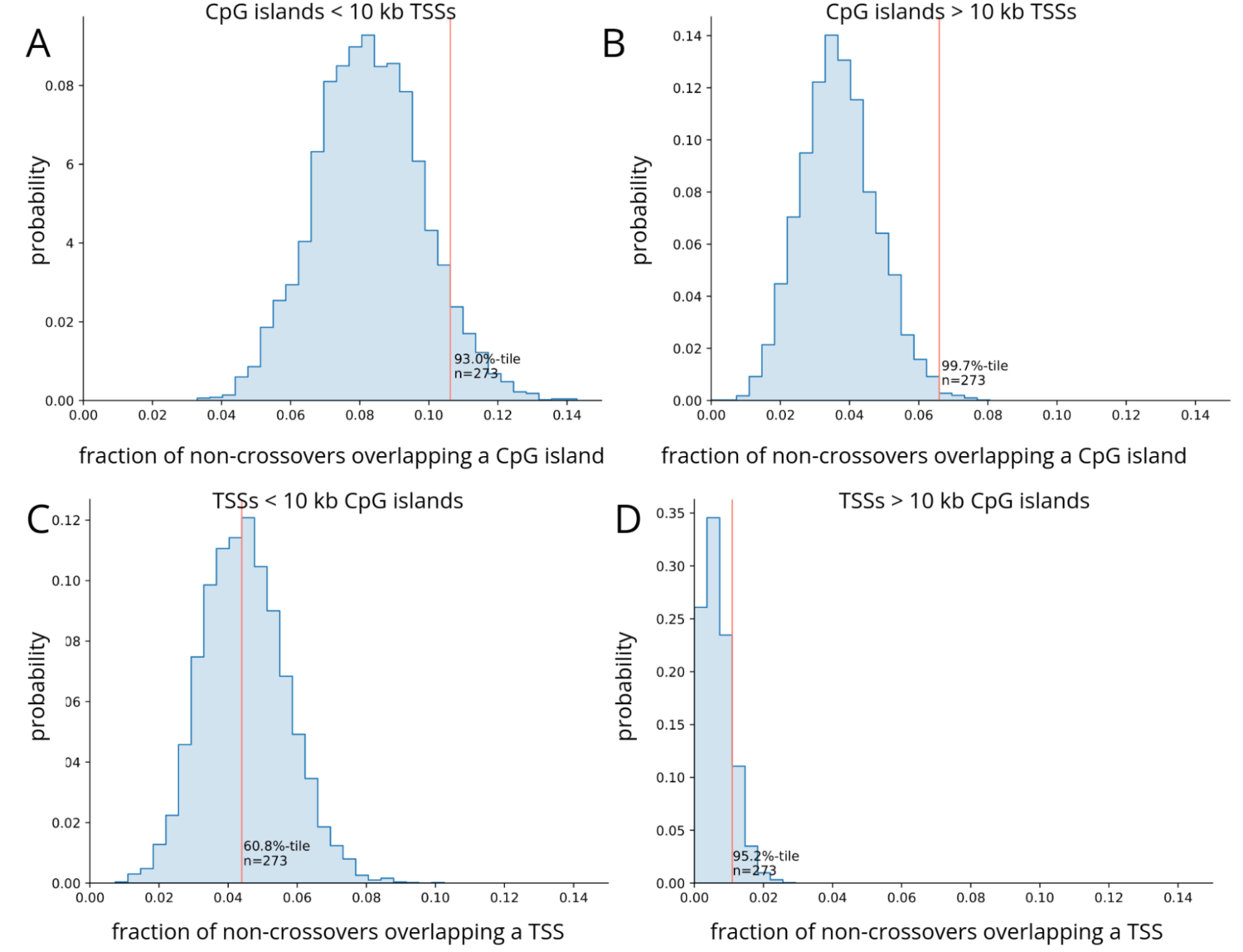

Supplement: S17 Fig — Fraction of non-crossover events overlapping (A) a CpG island at most 10 kb away from a TSS, (B) a CpG island farther than 10 kb from a TSS, (C) a TSS at most 10 kb away from a CpG island, and (D) a TSS farther than 10 kb from a CpG island. To account for the difference in width between CpG islands and TSSs, we considered that a non-crossover event overlaps a CpG island (A and B) and a TSS (C and D) if it is closer than 100 bps or 500 bps, respectively. The observed overlap is shown by red vertical lines. The overlap expected by chance, shown as a histogram, was obtained by randomly shuffling all crossovers events 5,000 times within 2.5 Mb of their original location, ensuring the shuffled location had a similar GC content and mappability (see Methods). (TIF) [file pgen.1011661.s017.tif]

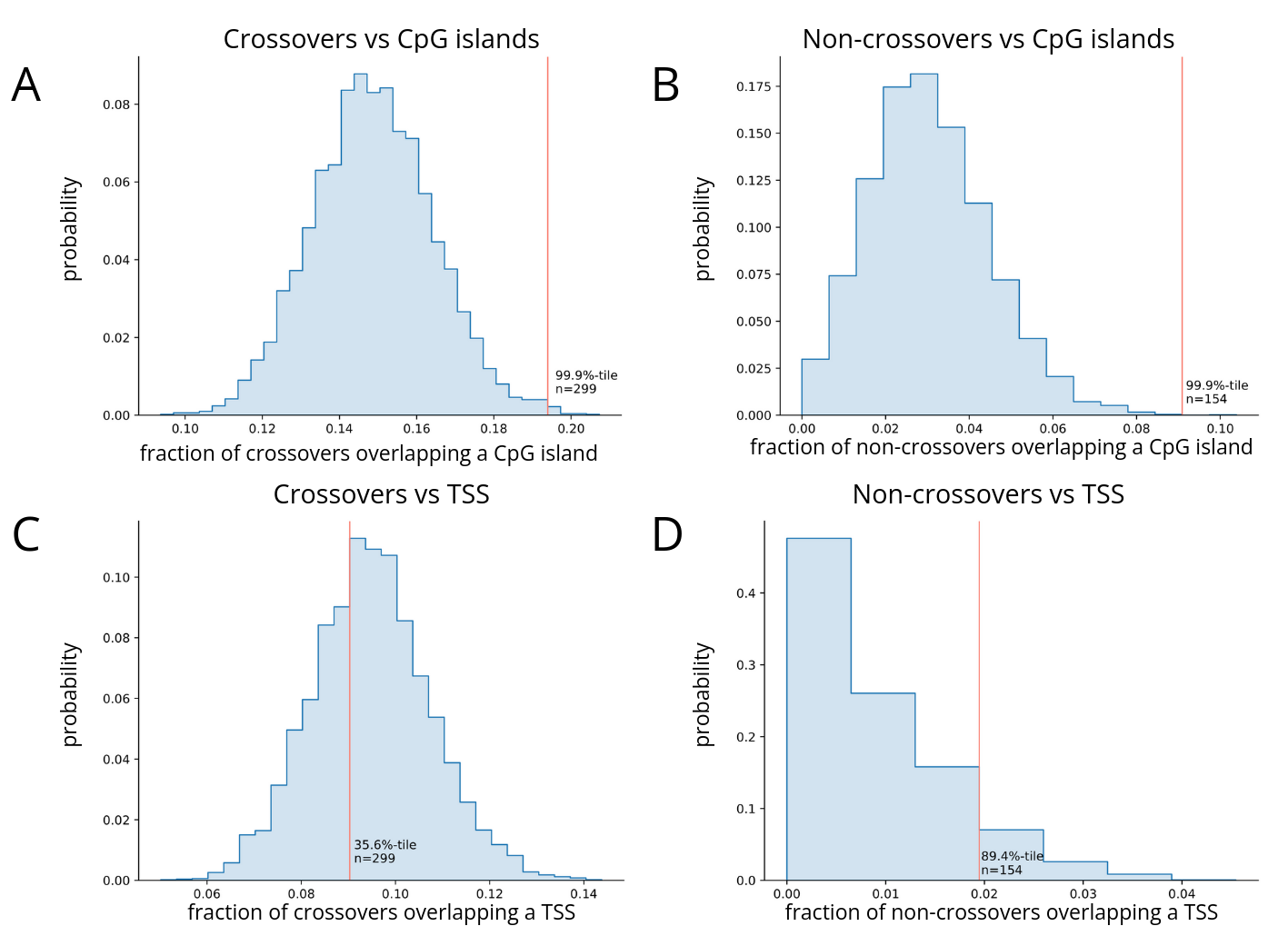

Supplement: S18 Fig — (A) Fraction of crossovers detected less than 100 bps from a CpG island. (B) Fraction of non-crossovers detected less than 100 bps from a CpG island. (C) Fraction of crossovers detected less than 100 bps from a TSS. (D) Fraction of non-crossovers detected less than 100 bps from a TSS. The vertical lines show the observed overlap. The overlaps expected by chance are shown as histograms, obtained by randomly shuffling 5,000 times all the events within a 2.5 Mb window on each side of their original location, matching for the GC content but not for the mappability (see Methods for details). (TIF) [file pgen.1011661.s018.tif]

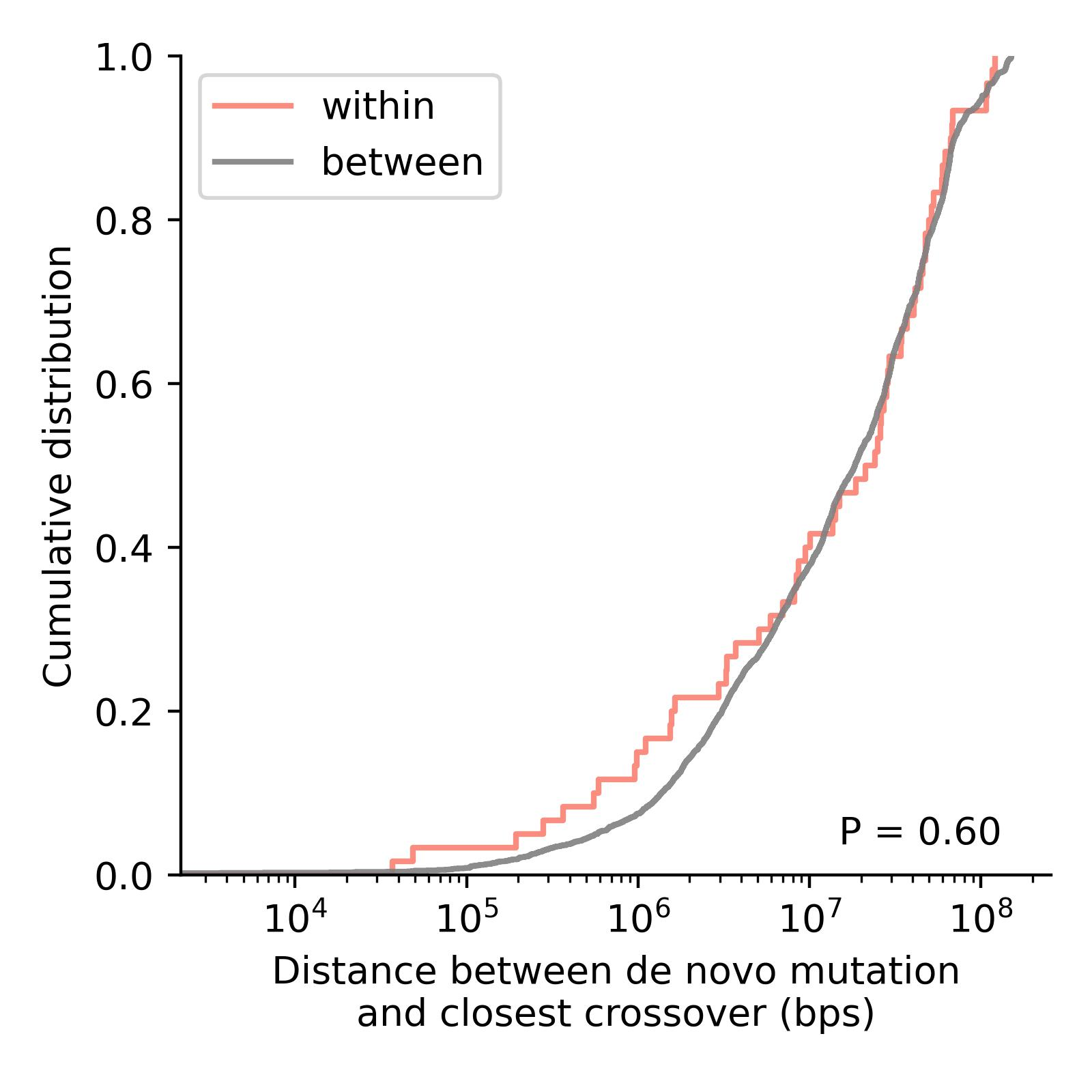

Supplement: S19 Fig — In red are the distances between events that occurred in the same germline (i.e., detected in the same proband and assigned to the same parental chromosome) and in gray between events that occurred in different germlines. The p-value was obtained from a Kolmogorov-Smirnov test. (TIF) [file pgen.1011661.s019.tif]

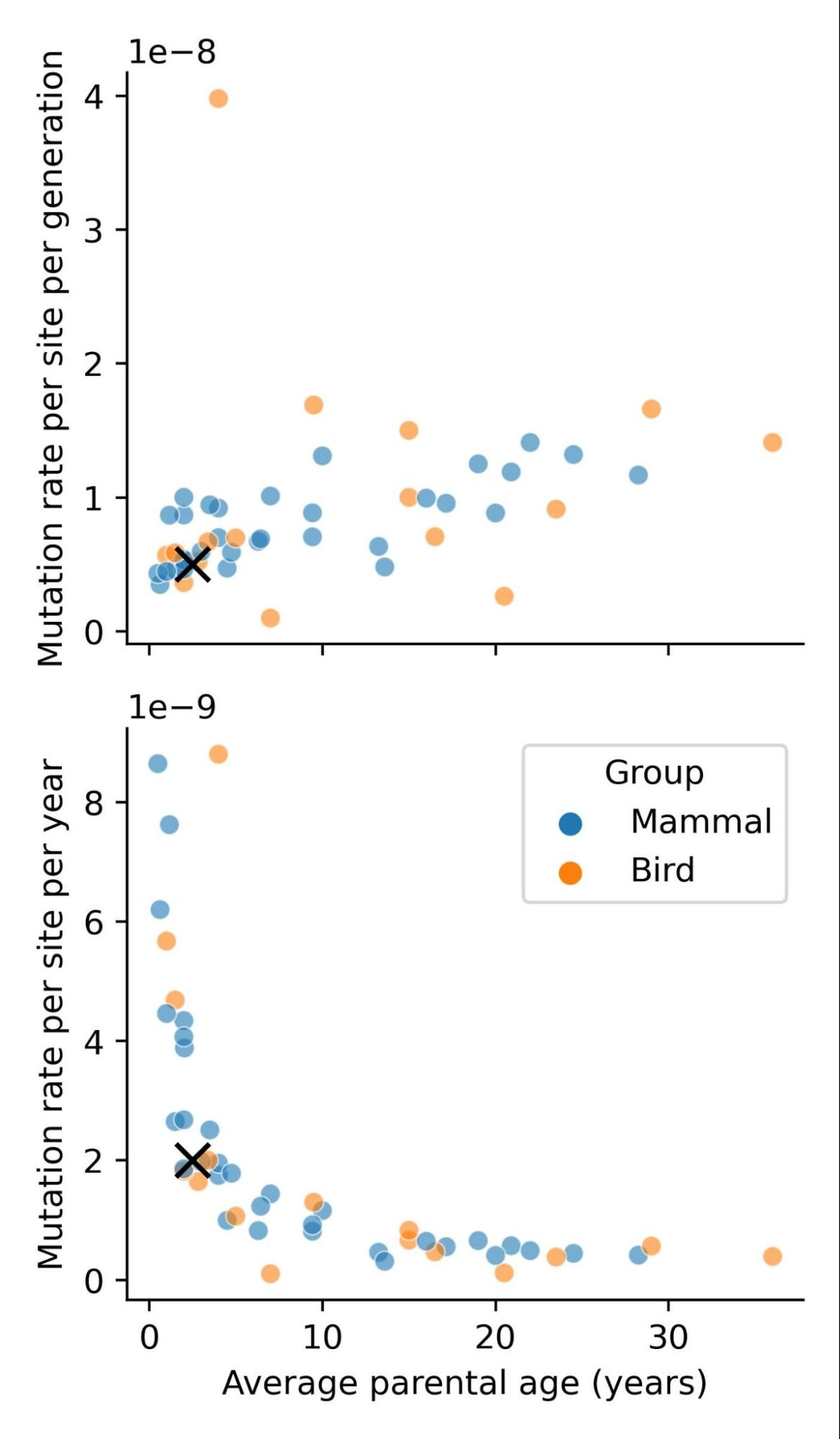

Supplement: S20 Fig — The average mutation rate per generation (top) and per year (bottom) is shown for 48 species of vertebrates. Data from [32] (their S2 Table); their estimates for mammals and birds are indicated by circles, while our point estimates for zebra finch are represented by crosses. (TIF) [file pgen.1011661.s020.tif]

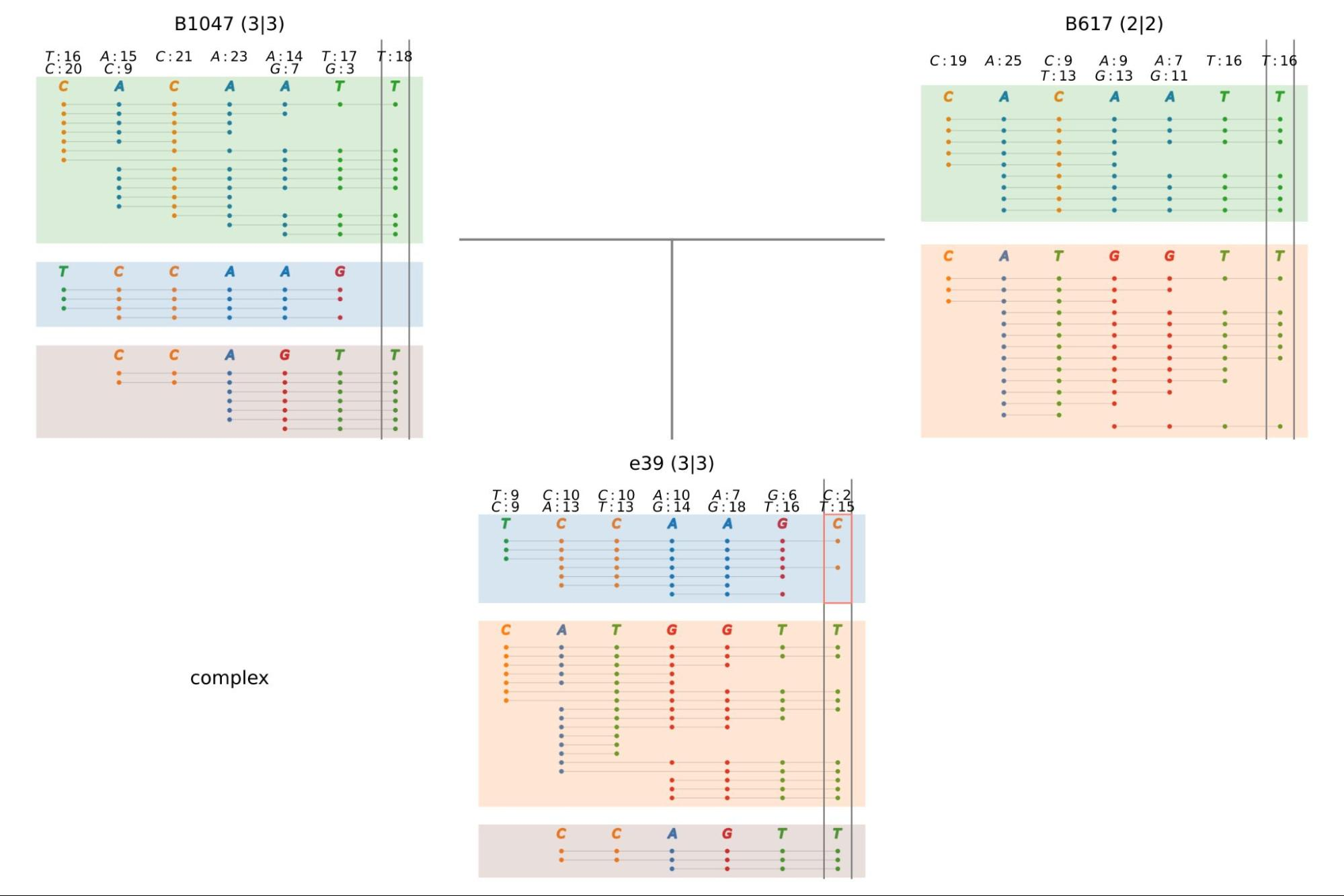

Supplement: S21 Fig — See S1 Fig for more details about how to interpret the figure. Note that the proband has no sequenced descendants, and thus only three individuals (instead of five) are shown. (TIF) [file pgen.1011661.s021.tif]

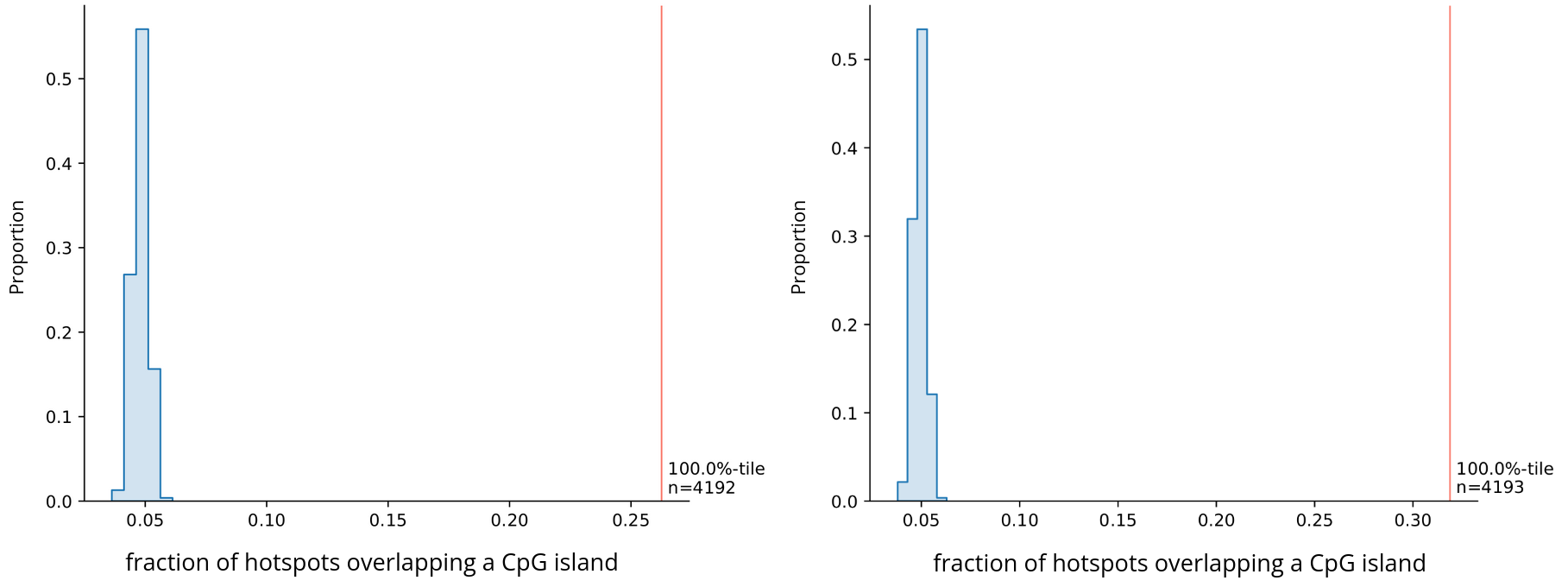

Supplement: S23 Fig — Fractions of hotspots within 100 bps of a CpG island, within 10 kb to a TSS (left), or greater than 10 kb from a TSS (right). The vertical red lines show the observed overlap. The overlap expected by chance is shown as a blue histogram, and was obtained by randomly shuffling all the events 5,000 times within a 2.5 Mb window on each side of their original location, matching for the GC content and ensuring a similar mappability (see Methods for details). (TIF) [file pgen.1011661.s023.tif]

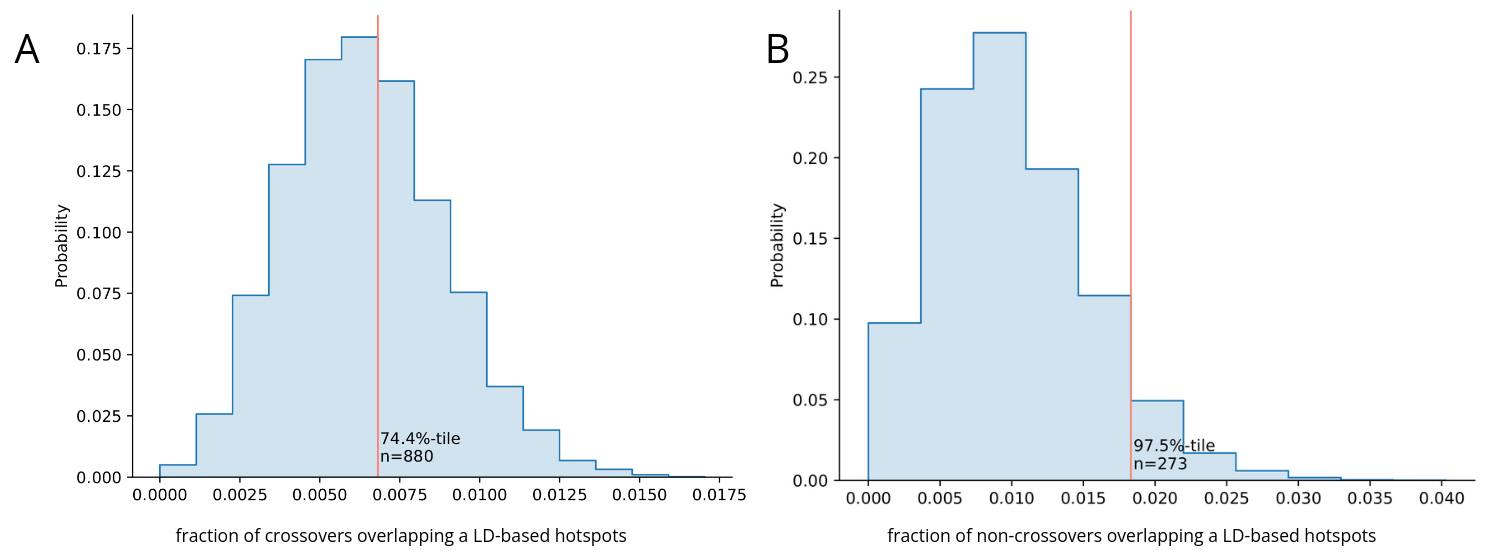

Supplement: S24 Fig — Fraction of recombination events within 100 bp from a LD-based hotspot. The overlap expected by chance is shown as a blue histogram, and was obtained by randomly shuffling all the events 5,000 times within a 2.5 Mb window on each side of their original location, matching for the GC content and ensuring a similar mappability (see Methods for details). (TIF) [file pgen.1011661.s024.tif]

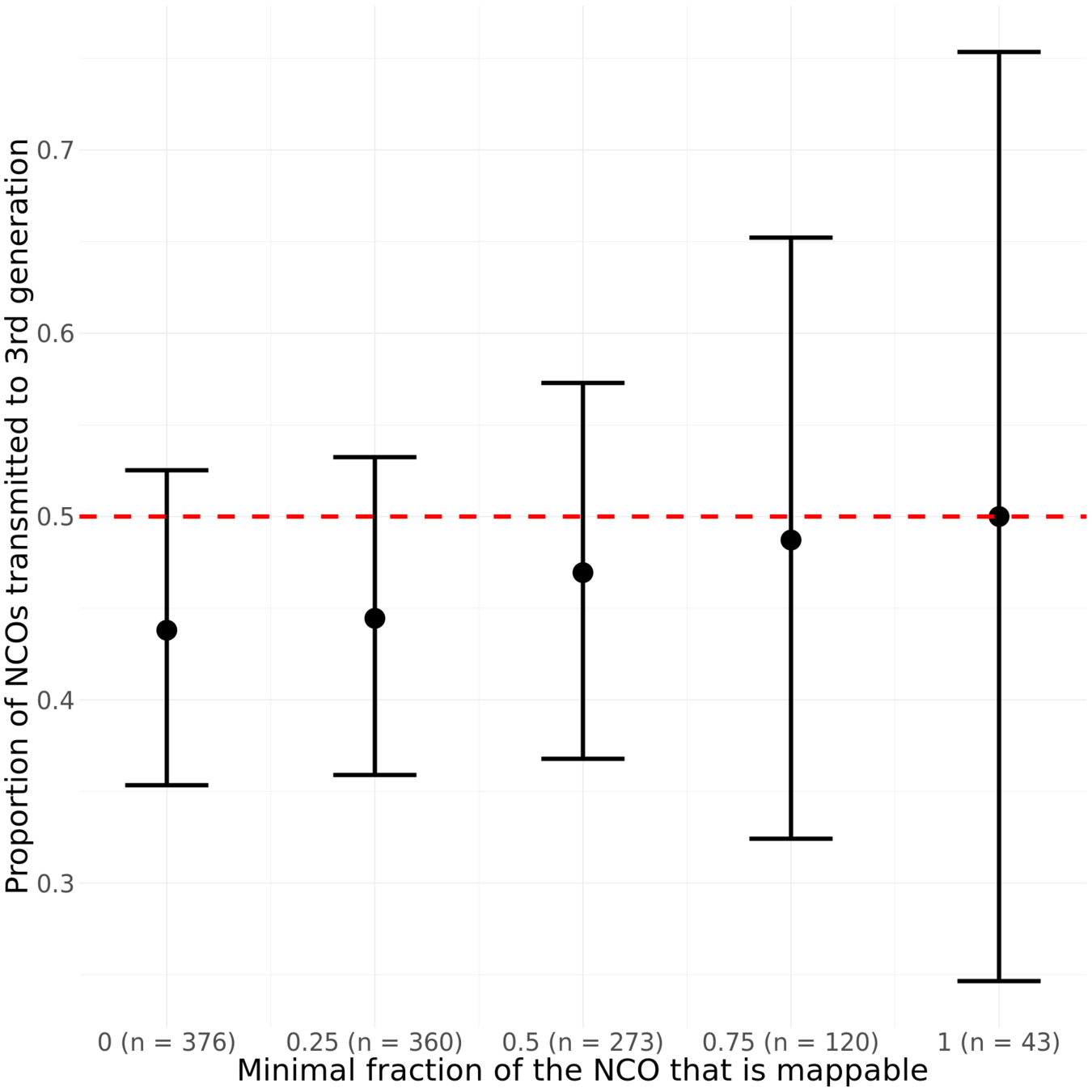

Supplement: S25 Fig — The sets were defined depending on the fraction of the length of the non-crossover interval that is mappable in the reference genome (see Method section “Detection of non-crossover events”). The number of non-crossovers in each set is in parentheses on the x axis label. The 95% confidence interval is represented by the black bars. The dashed red line denotes the expectation of 0.5 (assuming perfect power to detect a heterozygous site). (TIF) [file pgen.1011661.s025.tif]

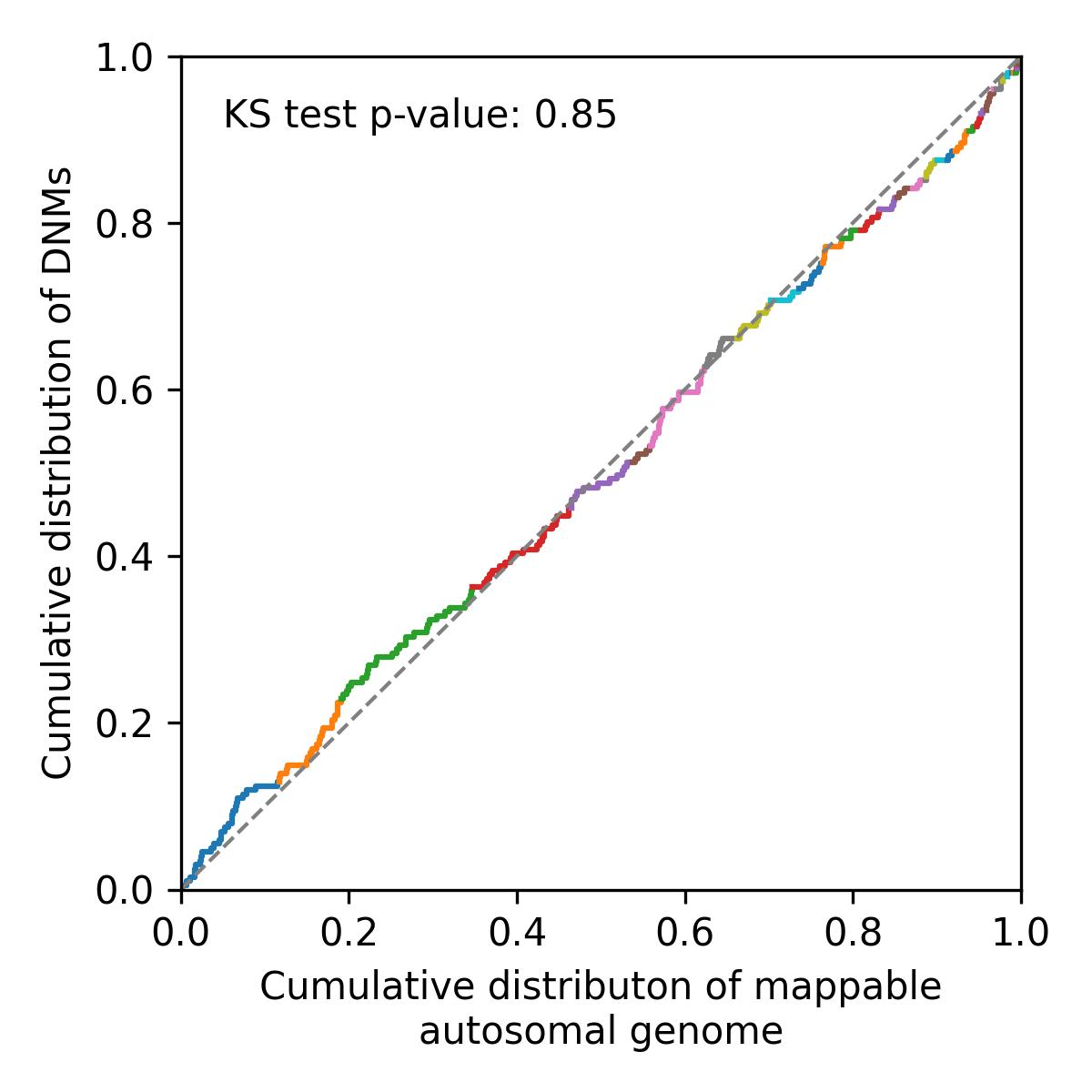

Supplement: S26 Fig — Colors correspond to chromosomes. The p-value is for a Kolmogorov-Smirnov test against a uniform distribution. (TIF) [file pgen.1011661.s026.tif]
