# Supplementary material for: Germline mutation rates and fine-scale recombination parameters in zebra finch
Source: PLoS Genet. 2025 Apr 15;21(4):e1011661. doi: 10.1371/journal.pgen.1011661 (PMC12047795; doi:10.1371/journal.pgen.1011661)
Supplement: S1 Text — (DOCX) [file pgen.1011661.s036.docx]

**Command lines**

Representative pseudo-code lines to reproduce the analyses. The more detailed scripts and READMEs can be found at https://doi.org/10.5281/zenodo.13696268.

| Whole genome sequencing alignment |
| --- |
| gatk HaplotypeCaller -R {ref} -I {bam} -O {vcf} --heterozygosity 0.01 \  -ERC BP_RESOLUTION -L {chrom} --pcr-indel-model NONE  gatk GenomicsDBImport --sample-name-map {input} -R {ref} -L {chrom} \  --genomicsdb-workspace-path {output}  gatk GenotypeGVCFs -R {ref} -V gendb://{input} -O {output} --heterozygosity 0.01 \  -L {chrom} |

| GATK variant calling |
| --- |
| gatk HaplotypeCaller -R {ref} -I {bam} -O {vcf} --heterozygosity 0.01 \  -ERC BP_RESOLUTION -L {chrom} --pcr-indel-model NONE  gatk GenomicsDBImport --sample-name-map {input} -R {ref} -L {chrom} \  --genomicsdb-workspace-path {output}  gatk GenotypeGVCFs -R {ref} -V gendb://{input} -O {output} --heterozygosity 0.01 \  -L {chrom} |

| Generation of mappability mask |
| --- |
| splitfa {Fasta} 150 \| split -l 20000000 --filter='gzip > \$FILE.gz'- kmers/{Chunk}    bwa aln -R 1000000 -O 3 -E 3 {fasta} kmers/{Chunk} > bwa/{Chunk}.sai     bwa samse {Fasta} bwa/{Chunk}.sai kmers/{Chunk} \| gzip > bwa/{Chunk}.sam.gz    gzip -dc bwa/*.sam.gz \| perl gen_raw_mask.pl > rawMask_150.fa    gen_mask -l 150 -r 0.5 rawMask_150.fa > mask_150.fa    python makeMappabilityMask.py mask_150.fa > {Mappability_mask.bed} |

| Detection of single-point *de novo* mutations |
| --- |
| python scripts/call_dnms.py -i {vcf} -t {trio} -c {chrom} |

| Assigning parent of origin of *de novo* mutations |
| --- |
| python scripts/classify_dnms.py {bams} -v {vcf} -m {start} |

| Inference of mutation signature activity |
| --- |
| Analyze.cosmic_fit({input_matrix}, {output_dir}, input_type="matrix") |

| Population structure and relatedness |
| --- |
| vcftools --gzvcf {input.vcf} --chrom-map {input.chrommap} --plink --out {params.outname1} --chr {wildcards.chromosome} --temp tmp/  plink --allow-extra-chr --geno 0.10 --chr-set 1 no-xy no-mt --indep-pairwise 50 5 0.2 --maf 0.05 --out {params.outname2} --file {params.outname1}  plink --chr-set 1 no-xy no-mt --allow-extra-chr --pca --file {params.outname1} --extract {params.outname2}.prune.in --out {params.outname3}  zcat {input.vcf} \| vcfsnps \| vcfbiallelic \| vcftools --vcf - --weir-fst-pop {input.pop1} --weir-fst-pop {input.pop2subset} --fst-window-size {params.wind_size} --fst-window-step {params.wind_step} --out {params.outfst_sub}  zcat {input.vcf} \| vcfsnps \| vcfbiallelic \| vcftools --vcf - --relatedness2 --out {params.outrelatedness} --exclude-bed {params.toexlude} |

| Diversity and effective population size estimates |
| --- |
| bedtools coverage -sorted -a {input.mappable} -b <(bcftools filter -g 5 {input.vcf} \| vcfsnps) > {output} |

| Ancestral alleles and mutation transition matrix |
| --- |
| #### test if allele is significantly major  vcftools --gzvcf {input} --freq --out out_ancestral/onesided_test_unrelated/{wildcards.chromosome}  sed '1d' out_ancestral/onesided_test_unrelated/{wildcards.chromosome}.frq -i  python scripts/binom_test_major_allele_exceed.py out_ancestral/onesided_test_unrelated/{wildcards.chromosome}.frq {output.all}  awk 'length($8)==1 && length($9)==1' {output.all} > {output.all}_tmp  mv {output.all}_tmp {output.all} -f  awk '{{if ($7<0.05) print}}' {output.all} > {output.filtered}  #### compute mutation transition matrix  cat {output.filtered} > {params.mut}  python scripts_compute_mut_mat.py {params.mut} {input.fasta} {output} |

| Variant phasing |
| --- |
| gatk VariantFiltration -V {input} -O {output.tmp} "  "--filter-expression 'QD < 6.0 \|\| MQ < 40.0 \|\| FS > 10.0 \|\| SOR > 4.0 \|\| "  "MQRankSum < -12.5 \|\| ReadPosRankSum < -8.0' --filter-name 'hard_filter' --verbosity ERROR;"  "vcftools --gzvcf {output.tmp} --remove-filtered-all --max-alleles 2 "  "--recode --recode-INFO-all --stdout \| bgzip > {output.vcf};"  "tabix -f -p vcf {output.vcf}  bcftools filter -g 5 {input} \| vcfsnps \| bgzip > {output.vcf}  tabix -f -p vcf {output.vcf}  extractPIRs --bam {params.bam_list} --vcf {input} --out {output.outfile}  plink2 --allow-extra-chr --update-parents {input.ped_info} --vcf {input.vcf} --make-bed --out {params.out_name}  shapeit -assemble --input-bed {params.bed} --aligned --input-pir {input.PIRs} -O {params.out} --effective-size {params.N} --rho {params.rho} --duohmm -W {params.windows} --output-log {params.log_dir}/{wildcards.chromosome} --force --thread {params.num_threads}  shapeit -convert --aligned --input-haps {params.file_in} --output-vcf {output} --output-log {params.log_dir}/{wildcards.chromosome}  vcftools --ldhelmet --vcf {input} --chr {wildcards.chromosome} --out vcftools_output/{wildcards.chromosome} --keep {params.tokeep} --temp {params.tmpdir} |

| LDhelmet analysis |
| --- |
| ldhelmet find_confs --num_threads {params.cpus} -w {params.windows} -o {output.confs} {input}  ldhelmet table_gen --num_threads {params.cpus} -c {input.confs} -t {params.theta} -r 0.0 .0000001 .000001 .000001 .00001 .00001 .0001 .0001 .001 .001 .01 .01 .1 .1 1.0 0.5 50.0 -o {output}  ldhelmet pade --num_threads {params.cpus} -c {input.confs} -t {params.theta} -x {params.coeff} -o {output}  ldhelmet rjmcmc --num_threads {params.cpus} -w {params.windows} -l {input.likelihoods} -p {input.pade} -b {wildcards.bkpty} --snps_file {input.haplotypes} -m {input.mutmat} --burn_in {params.burn_in} -a {input.ancestral} --pos_file {input.positions} -n {params.iterations} -o {output}  ldhelmet post_to_text -m -p {params.quantile_1} -p {params.quantile_2} -p {params.quantile_3} -o {output} {input} |

| Identification of hotspots |
| --- |
| bedtools intersect -a {input.genome} -b {input.rec} -wo \| compute_rrate_windows.sh > {output}  bedtools map -a {input.windows} -b {input.recrate} -c 4 -o mean > {params.meanrate}  python scripts/relative_recomb_rate.py {params.meanrate} > {output} |

| Identification of CpG islands |
| --- |
| cpgplot -sequence ${REF} -outfile ${CPGPLOT_OUT} -window 50 -minlen 250 \\  -minoe 0.6 -minpc 50 -graph png -outfeat ${CPGI.GFF} -plot No  python scripts/HMM.py -s {chrom} |

| Detection of crossovers |
| --- |
| python scripts/call_crossovers.py -i {vcf} -f {fam_ped} -m {min_info_sites} -p {out_pdf} -v {prefix} -o {out_bed} |

| *Freebayes* variant calling |
| --- |
| bamaddrg {bams} -R {region} -c \  \| freebayes -f {ref} --stdin -g 10000 --min-mapping-quality 30 \  --min-base-quality 30 --use-best-n-alleles 4 \  \| vcfallelicprimitives -kg \  \| bgzip > {vcf} |

| *Bcftools* variant calling |
| --- |
| bcftools mpileup -b {bams} -f {ref} --max-depth 150 --min-BQ 30 \  --min-MQ 30 -r {region} -a AD,ADF,ADR,SP,INFO/AD,INFO/ADF,INFO/ADR -Ou \  \| bcftools call -Ou -mv \  \| bcftools filter -s LowQual -e '%QUAL<20' -Oz -o {vcf} |

| Detection of non-crossovers events (including extra filtering steps) |
| --- |
| zcat {input.vcf} \| grep -v "\.:\.:\.:\." \| python {params.script1} -s {input.depth} -u {params.upper} -l {params.lower} \| bgzip > {params.tmpfile}  bcftools query {input.vcf} -f '%CHROM\t%POS\t%REF\t%ALT\t[%SAMPLE=%GT\t%RO\t%DP\t%AD\t]\n' -S {input.GdOff_ind} \| grep "=\." -v \| python3.9 {params.script2} \| awk '{{print $1"\\t"$2"\\t"$2}}' \| uniq \| bgzip > {params.postoremove}  bcftools view -T ^{params.postoremove} -o {output} {params.tmpfile} -O z  bcftools +mendelian {input.vcf} -T {input.trio} -d \| bgzip > {output.outvcf}  bcftools query -S {input.gd_off} {input.vcf} -f '%CHROM\t%POS\t%REF\t%ALT\t[%SAMPLE=%GT\t%RO\t%DP\t%AD\t]\n' \| python {params.script} \| grep "\./\." -v \| cut -f1-2 > {params.tmp_pos}  tabix -h {input.vcf} -R <(grep {wildcards.chromosome} {input.mappable} \| awk '{{if ($2==0) print $1"\\t""1""\\t"$3; else print $1"\\t"$2"\\t"$3}}') \| bgzip > {output.outvcf}  python scripts_noncrossovers_detection.py {input.ped_info} {input.vcf} {output.maternal} {output.paternal} {params.maternal_dir} {params.paternal_dir} |

| Phasing of non-crossovers |
| --- |
| whatshap phase --reference {ref} --ped {ped} --use-ped-samples {vcf} {bams} -o {output} |

| Conversion tract length estimation |
| --- |
| Rscript script_lileklyhood.R {params.dir_in} {output} |

| Estimating the number of non-crossovers in a meiosis |
| --- |
| # R code to sample 10,000 windows from the exponential distribution of rate 1/15  n <- 10000  rate_15bp <- (1/15)  samples_15bp <- rexp(n, rate_15bp)  # head(samples)  rounded_samples_15bp <- round(samples_15bp)  write.table(rounded_samples_15bp, file = "sampling_from_exp_distrib_15bp.txt", row.names = FALSE, col.names = FALSE)  ## bash loop for 100 iterations  for i in {{1..100}}  do  cat {input.sampling} \| awk '{{print "{wildcards.chromosome}""\\t""1""\\t"$1+1}}' \| bedtools shuffle -i - -g {input.chr_size} -chrom \| \  bedtools sort \| uniq \| bedtools intersect -a - -b {input.info_sites} -wao \| awk '{{if ($1==$4) print}}' \| cut -f1-3 \| uniq -c \| awk '{{print $2"\\t"$3"\\t"$4"\\t"$1}}' > {params.suffix}_${{i}}.bed  done |
