# Supplementary material for: Germline mutation rates and fine-scale recombination parameters in zebra finch
Source: PLoS Genet. 2025 Apr 15;21(4):e1011661. doi: 10.1371/journal.pgen.1011661 (PMC12047795; doi:10.1371/journal.pgen.1011661)
Supplement: Attachment — Submitted filename: Prentout_et_al_Reply.pdf [file pgen.1011661.s037.pdf]

We thank the reviewers for their insightful comments, which led us to add a couple of analyses and in particular to discover a set of putative class II crossovers that we had not noticed previously. We have addressed each point raised and believe that the revisions have improved the clarity and quality of our manuscript.

We also note that during the revision process, we found an error in our code. We have now fixed it, and estimates of the mean conversion tract length and the non-crossover-to-crossover ratio have changed somewhat; they are now even closer to those estimated in mice (Cole et al, 2010; Li et al, 2019).

Below please find a description of additional changes.

REVIEWERS' COMMENTS:

Reviewer #1:

In this study, Prentout and colleagues generated whole genome sequences from 74 zebra finch individuals to: (i) detect sex-specific de novo mutations from pedigrees, (ii) infer the developmental timing of germline mutations, (iii) estimate mutation rates, (iv) detect crossover and non-crossover events, (v) analyze the distribution of recombination events along the genome considering their genomic characteristics, and (vi) estimate parameters of gene conversion. While the study provides new genomic resources for the community, the authors should emphasize how their work distinguishes itself from previous studies.

It will be nice to acknowledge already in the introduction section previous research on recombination rates in birds using both cytological and pedigree approaches (see: McAuley et al. 2024 MBE; Tan et al. 2024 Heredity; Bascón-Cardozo et al. 2024 Genome Biol Evol; Robledo-Ruiz et al. 2022 Gigascience; Del Priore et al. 2020 PLOS One). Some of these studies have already reported male-biased recombination at chromosome ends and higher recombination rates in micro-chromosomes compared to macro-chromosomes. This pattern is expected to ensure obligatory chiasmata for proper chromosome segregation during the first meiotic division. As for the germline de novo mutations, recent reports have been published for zebra finch (Liang X et al. 2024 Commun Biol). It will be desirable if authors can distinguish their results from previous work.

We apologize for missing some relevant papers. With one exception (Bascón-Cardozo et al 2024), these studies focus on broad-scale recombination patterns. They report increased recombination on micro-chromosomes, and, depending on the species, that rates are male-biased (Robledo-Ruiz et al, 2022; Tan et al, 2024), female-biased (McAuley et al, 2024) or unbiased (Zhang et al, 2024). In turn, Bascón-Cardozo et al (2024) build a linkage disequilibrium-based map for the Eurasian blackcap and examine its relationship to genomic features. We have expanded the introduction (lines 86-87, 95, 100) and added

a sentence to the results (lines 293-298) to better situate our findings in the context of these papers.

Notably, our study adds to this body of work by identifying a large set of well-resolved crossover and non-crossover events. Analyses of these events reveal a number of new features of bird recombination, including: (i) documenting the absence of sex differences in the genomic distribution of recombination events in zebra finches, as well as providing (ii) the first direct estimate of the fraction of crossovers and non-crossovers that occur at CpG islands, (iii) the first estimate of the non-crossover-to-crossover ratio, taking into account power differences, and (iv) the first estimate of the mean conversion tract length in an avian species.

One of the references provided by the reviewer, Liang et al. (2024), was published in *Communications Biology* on October 2nd, after we had posted our manuscript to BioRxiv and submitted it to *PLOS Biology*. Therefore, we did not have the opportunity to discuss it and compare our results. We have expanded the introduction accordingly (lines 123-128). Their work and ours agree on the mutation rate per generation (6.3×10^{-9} and 5.0×10^{-9} per bp, respectively); both studies also find no detectable association between recombination and germline mutation rates, as we now note. Furthermore, Liang et al. (2024) estimate a paternal bias in germline mutation, albeit a stronger one (3.9) than that inferred in our study (1.7). We provide a possible explanation for this difference based on the younger average parental age in our study (lines 266-269). We note that this study also reports a surprisingly high transition-to-transversion ratio among *de novo* mutations (3.4), which we do not find among DNMs (ours is 1.7) or low-frequency polymorphisms (SupFig S3), nor do other studies to our knowledge.

Despite these overlaps, our study offers novel insights into germline mutation in birds, specifically: we find that (i) mutation rates are remarkably similar in macro- and micro-chromosomes, despite notable differences in genomic features between these chromosome types, (ii) the COSMIC mutational signatures most active in the mammalian germline (SBS5 and SBS1) also predominate in birds, suggesting that mutational processes may be phylogenetically conserved, and (iii) the classification of germline mutations by their developmental timing reveals a window of avian development with no detectable sex bias in mutation, mirroring observations in mammals (Harland et al, 2017; Jonsson et al, 2017; Lindsay et al, 2019).

Reviewer #2:

In this manuscript, the authors utilize sequencing data from several two- and three-generation zebra finch pedigrees to comprehensively analyze the characteristics of mutation and meiotic recombination. While some of the results reiterate and confirm existing knowledge (e.g., recombination rate, distribution patterns, absence of heterochiasmy, differences between macro-

and microchromosomes), this study provides a valuable resource for future comparative analyses. It offers the first estimates of important characteristics in zebra finches, such as the NCO/CO ratio and conversion tract length, and could be of interest to the researchers in bird genomics and the evolution of recombination.

The methods are clearly and thoroughly described allowing to replicate the study. All results are properly statistically supported. The authors accurately handle data using a conservative approach and rigorous data filtering to avoid false positives and other sources of error and ensure accuracy and reliability.

Please find below my comments on the presentation and interpretation of the results.

1) While I generally agree with the conclusion regarding the stability of certain parameters across taxa, I find the claim of stabilizing selection to be somewhat overgeneralized.

First, some of the examined features indeed seem to be mechanistically constrained by molecular mechanisms and chromosome segregation requirements. However, at least the recombination rate is known to vary significantly across species and taxa. There's been extensive discussion in the literature about whether this variation is adaptive or neutral (e.g., Stapley et al., 2017). The comparison of one bird species with mammalian taxa doesn't seem sufficient to infer selection regimes.

Moreover, birds exhibit a huge variation in recombination rates, with differences of up to 2-fold, which primarily affects macrochromosomes (e.g., Semenov et al., 2018), and the zebra finch has one of the lowest rates. Additionally, the variation in both the direction and extent of heterochiasmy (e.g., McAuley et al., 2024) adds further complexity to the picture. Therefore, while comparing recombination rates in zebra finch macrochromosomes with those in mammals may be appealing, making broad evolutionary conclusions from such comparisons appears overly selective.

Thus, I recommend authors to revise their generalizations about stabilizing selection.

We agree with this concern, and have revised the manuscript and changed the title accordingly.

In particular, we appreciate that recombination rates vary among birds and are generally higher than in mammals. In our view, it is striking that mean rates per bp across a huge range of taxa and karyotypes vary less than ~4-fold (Stapley et al. 2017), but such a discussion is beyond the scope of this study.

What we wish to highlight instead is that despite hundreds of millions of years of evolution and the absence of PRDM9 in birds—and thus using a completely different mechanism to specify DSB locations—many of the fine-scale recombination parameters that we estimate in zebra finch almost identical to those in mice, including crossover to

non-crossover ratios, GC biased gene conversion rates, and the estimated mean conversion tract length. In our view, mechanistic constraints by molecular mechanisms are not a satisfactory answer, given that we know it is possible for these values to differ drastically, as some do in yeast species, *A. thaliana* or Drosophila species for example, and given the existence of mutation and recombination modifiers that segregate within populations. Thus, selection must be weeding out changes to the genes involved in these processes. Since many features, such as tract length, are quantitative traits that could increase or decrease, it seems plausible that their conservation is reflective of stabilizing selection. We have now made our reasoning clearer in the Discussion, and tempered our claim.

2) Based on Fig. S5A, it seems that several macrochromosomes have more than one CO per chromatid. It would be worth analyzing the characteristics of these double crossovers - the distance between them and interference (if data allow). Identifying closely spaced double crossovers could provide insight into whether birds possess a second, non-interfering CO resolution pathway, which is currently unknown.

We thank the reviewer for this helpful comment. We found 293 instances of a chromosome with more than one crossover in a given meiosis. Of these, 95 crossovers (8% of the total 1,174 crossovers) involve two crossovers within 1 Mb of each other. These events are very well supported, in that we require ten informative markers on each side of a crossover, among other stringent filters. We therefore believe that most are real crossover resolutions. These events are equally distributed between maternal (45 events) and paternal (50 events) meioses (*p*-value from an exact binomial test = 0.41). As suggested by the reviewer, they are plausibly the result of a non-interfering crossover resolution pathway, i.e., class II crossovers. Indeed, such class II crossovers are estimated to represent 5-10% of crossover events in mice (Holloway et al, 2008). However, we cannot exclude the possibility that a subset are long gene conversion tracts. Previous studies in humans and baboons report that up to ~2% of gene conversion tracts can exceed 10 kb, potentially extending several hundreds of kb long (Halldorsson et al, 2016; Wall et al, 2022; Charmouh et al, 2024). Such events could also arise from complex resolutions with template switching (as described in Williams et al, 2015; Halldorsson et al, 2016; Wall et al, 2022). We now report these results and discuss these possibilities on Lines 381-395.

We also investigated crossover interference by comparing the distribution of distances between crossovers occurring within the same meiosis (“within”) to the expected distribution derived from events occurring in different meiosis (“between”). We find that the “within” distribution is shifted towards longer distances (Figure R1), as expected under crossover interference. Interestingly, the “within” distribution appears to be bimodal, with an enrichment of events separated by shorter distances (~10^5 bps), likely reflecting the presence of a subset of class II crossovers. We have included this result in

the Main Text (Lines 382-386) along with a Supplementary Figure (S11) and a Method section (Line 963-967).

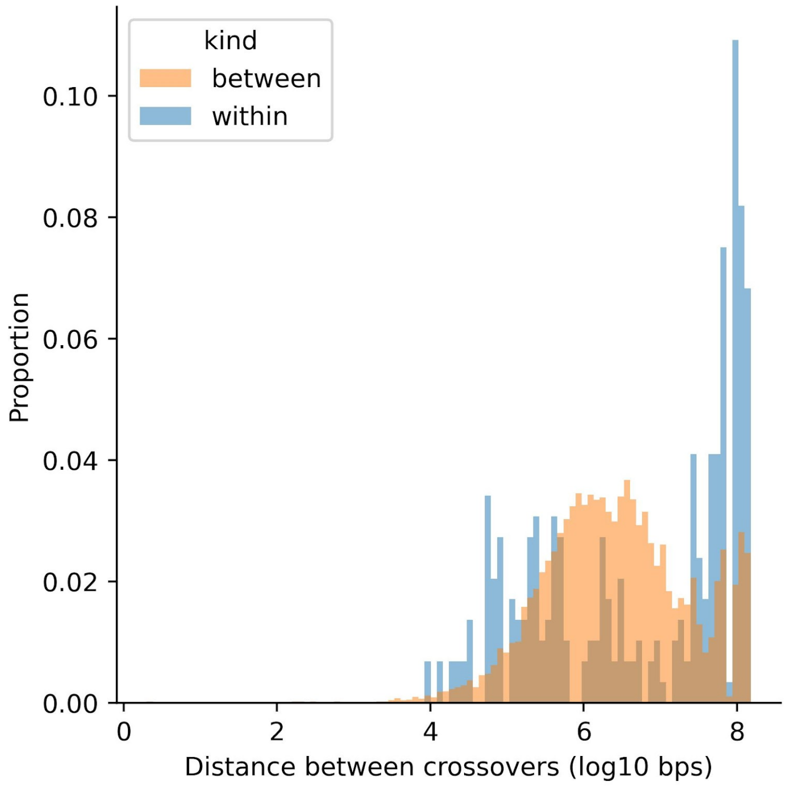

<u>Figure R1.</u> **Distribution of distances between the closest pairs of crossover events, for comparisons of events that occurred within the same meiosis ("within", blue) or a different meiosis ("between", orange).**

Finally, we investigated the consequences of chromosomal inversions on the distribution of crossovers. As expected, our analysis showed a clear suppression of crossover events within the putative inverted region, for individuals that we infer to be heterokaryotic. We added a method section (Lines 1050-1074) describing the karyotyping of individuals for three well-characterized autosomal inversions, as well as a Results section (Line 376-380) and a corresponding Supplementary Figure (S10) to present these findings.

3) Could the authors clarify why microchromosomes in Fig. S7B have more repetitive elements? As far as I know, bird microchromosomes generally have more genes and fewer repeats than macrochromosomes.

The observed higher repeat content in very small micro-chromosomes is consistent with findings reported by the Vertebrate Genome Project for zebra finch (see Kim et al, 2022). Specifically, most "dot" chromosomes (*i.e.* micro-chromosomes smaller than 5 Mb) have

both higher GC-content and higher repeat content compared to larger micro-chromosomes. This pattern has also been described in the chicken genome (Huang et al, 2023).

4) Are the detected de novo mutations evenly distributed across mappable regions? Could the distribution of mappable regions on the chromosomes affect the results?

The DNMs that we detect appear to be evenly distributed across the mappable autosomes (Figure R2), suggesting there is relatively low mutation rate variation across the genome in zebra finches. Also in support of rates being relatively uniform, we estimated similar rates and spectra in regions in the macro- vs. micro-chromosomes (Figure 1B-C).

The distribution of mappable regions could influence our estimation of the genome-wide autosomal mutation rate if there were a systematic difference in mutation rates between mappable and non-mappable regions. In that regard, we note that mappable regions are distributed fairly homogeneously along the chromosomes, with the exception of telomeric regions of microchromosomes. Given this homogenous distribution, other chromosomal features with known chromosome-scale variation that can affect mutation rates (e.g., replication timing, nucleotide content) seem unlikely to bias estimates in mappable vs. non-mappable regions. Having said that, recent pedigree studies in humans based on high accuracy, long-read sequencing technologies reveal mutation rates to be elevated in low-complexity regions (Porubsky et al. 2024). Whether this observation extends to avian species remains an open question, requiring further investigation with sequencing approaches that allow a more comprehensive analysis of the genome.

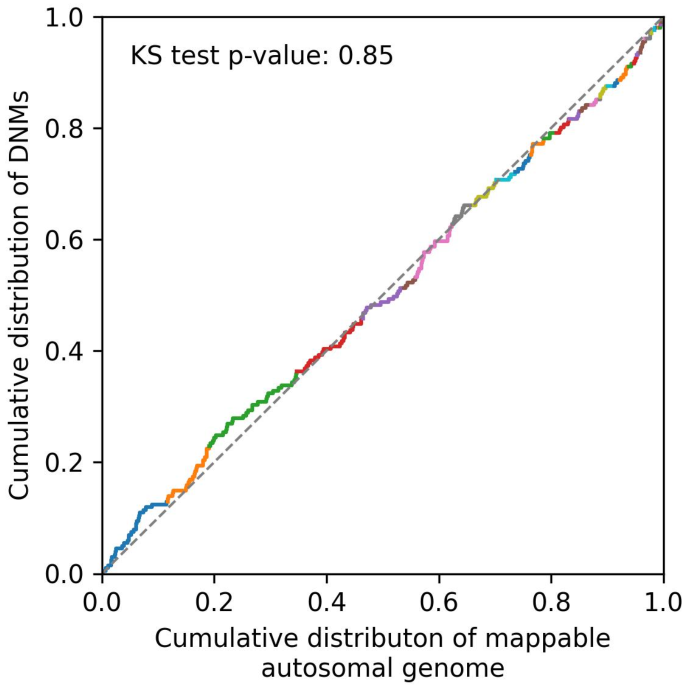

<u>Figure R2.</u> **Distribution of DNMs along the mappable autosomal regions. The p-value of a Kolmogorov-Smirnov test against a uniform distribution is annotated.**

5) Please discuss your findings in the context of just published paper on de novo mutations in zebra finch (Liang et al., 2024).

As noted in the response to reviewer 1, this paper appeared after ours, so we did not have the opportunity to comment on it previously. We now do so.

There are also some minor points:

6) Please revise the reference list. For example, the citation of Soler et al. (2021) from p.4 is missing

This error has now been fixed.

7) Although the manuscript is generally clearly written, some phrases are difficult to follow. I would recommend revising the text for clarity. There are also some typos in the main text and supplementary materials (e.g., "rappability" in Fig. S5; "great weed warbler" on p. 3).

Thank you for noting these typos, which have been corrected.

8) Line numbers would help a lot in reviewing.

We apologize for this oversight. Somehow the line numbers disappeared when we extracted the pdf. This has now been fixed.

Reviewer #3:

This paper by Prentout et al investigates mutation rates and recombination (through crossovers and non-crossovers) in 74 sequenced zebra finch families (4 full-sib families and 4 trios = 80 informative meioses).

First, the authors investigated de novo mutations (DNMs) across ~50% of the genome that was informative, identifying 202 DNMs. They classified a number of these DNMs as germline or post-zygotic (i.e. arising in early development), showing that the latter had higher levels compared to humans, but similar to mice and cattle. Males had higher mutation rates than females in the germline. Interestingly, they find no difference in DNM rate between micro- and macro-chromosomes.

Second, the authors investigated meiotic recombination in 54 meioses, identifying 1,174 autosomal crossovers to high precision by tracking inheritance between three generation pedigrees to identify sites of COs and NCOs. They confirmed similar crossover counts between females and males. They determined the mean recombination rate was 2.28cM/Mb (with variation between macro- and micro-chromosomes). They constructed an LD-based map in LDhelmet. They also investigated NCO events using several different approaches, identifying 275 events over 36 meioses; these events were more evenly distributed across macrochromosomes than CO events. The authors investigated parameters of gene conversion events, identifying the the GC tract length can be ~23 to 35 bps on average. When adjusting the NCOs to the ratio they expect to find, they suggest that the NCO:CO ratio is 6.7.

This is a well-written paper, and I enjoyed reading it. The analyses are well described, meticulous, and reproducible - it is clear what the authors have done and why, and I was convinced that a lot of care has been taken to ensure that the results are reliable and appropriately interpreted. Nevertheless, I did have some comments.

One of the main claims of the paper at the end of the abstract is that the findings in this paper suggest that mutation and recombination are evolving under stabilising selection, because the findings are very similar to those in mammals. I agree that this is possible, but the paper gives very little development to that idea - does conservation truly indicate stabilising selection? If the authors want to assert this claim, there needs to be a more developed discussion. Rather than saying that better sequences should make this possible, please explain how and why, and consider whether this claim should be toned down.

The title strongly implies that this paper includes analyses in both mammals and zebra finches. I think care should be taken to ensure the reader knows that this paper has a primary focus on

zebra finches, and that the mammal part comes from comparisons with previous findings. Maybe something better than "Mutation and recombination parameters in zebra finch show conservation with mammals" or something along those lines?

We thank the reviewer for this suggestion, and have now changed the title as suggested.

Page 10/32: I didn't immediately understand the comment on Page 32 "(i.e., polymorphic sites… three chromosomes)" - I guess that the authors mean the same site in 3 individuals i.e., 3 chromosomes out of 54? Why was this threshold chosen (was it to overcome the DNM rate?)?

We apologize for the lack of clarity and have now edited the sentence to clarify it. We defined low frequency polymorphisms as those carried by at most three chromosomes in the sampled population (i.e., 3 out of 54, as the reviewer noted). These low frequency (young) alleles are expected to most closely reflect germline mutational processes, as they are typically recent and thus are less influenced by selection or biased gene conversion. In principle, singletons are expected to most closely resemble de novo mutations but, relative to other frequencies, they are also enriched for sequencing errors. We therefore relaxed the frequency threshold to include variants observed in at most three chromosomes (~5% frequency in our sample).

Page 10/32: COSMIC analysis. This is not well explained - it would be good to add more general information about what this analysis is doing. Please also define what a mutational signature is, and define what SBS1, SBS5, etc. is, as it is not clear to someone unfamiliar with this type of analysis.

We apologize for not having sufficiently introduced the concept of mutational signatures. We now provide a more detailed introduction (lines 234-243).

Page 12: was the recombination dataset also estimated from the ~50% "informative" genome, or across the whole genome?

The informative markers that we use to call crossover events are indeed from 50% of the genome, but from them, we learn about crossovers that occur throughout the genome. Specifically, given that a crossover is visible through an exchange of flanking markers on either side (in practice, we require at least 10), if a crossover happens in a masked section of the genome, we will still identify it having occurred, only its location will be poorly resolved. More generally, using a pedigree approach based on whole-genome sequencing, the density of informative markers has little effect on the number of crossovers identified, but rather shapes the interval size within which the location of crossovers can be resolved.
For non-crossovers, the situation is different, as we can only detect events that occur in the "informative genome", which directly affects the density of informative sites. We corrected for this effect, as described in lines 528-530, and noted explicitly that this is

based on the assumption that the regions in which we call events are no different from those in which we cannot (lines 1044-1045).

Related to that - it would be good to know the total number of polymorphic sites investigated for each of the analyses.

The detection of crossover and non-crossover events was based on 18,872,093 and 13,995,830 informative sites, respectively. We have now included these numbers in the main text (Lines 886 and 944)

Page 13: Regarding the difference in cM/Mb with the previous studies, Fig 2 demonstrates quite nicely that zebra finches have recombination events strongly skewed to the telomeres, so its possible that those lower marker densities did not have enough coverage in these regions to call crossovers. The authors state that these previous studies likely "missed a number of crossover events", perhaps this is the reason why?

We agree that low marker density in telomeric regions of the macro-chromosomes can lead to an underestimation of the recombination rate. We had mentioned this possibility in the main text (Lines 322-324: "Moreover, previous estimates were based on few genetic markers (~900 and ~2,000, respectively), so may have missed a number of crossover events, even on larger chromosomes."). Another likely source of under-estimation in previous studies is their exclusion of very small micro-chromosomes (see lines 318-322).

Pages 17 - 19: The methylation data was only obtained from males, which are likely to undergo large genome restructuring around meiosis. Do the authors know if the methylation patterns are corresponding between the sexes, and would it make sense to look only at male crossovers here as well? Perhaps it does not affect the conclusions of the paper, but it would at least be appropriate to acknowledge a potential effect of sex or differences between the sexes here.

We thank the reviewer for raising this question. To our knowledge, it is not known whether methylation patterns are the same in meiosis in the two sexes.
We redid the analysis using only the recombination events identified in males. As shown in Figure R3, the results are qualitatively the same: the fraction of crossovers that overlap a CpG island is slightly lower, but remains significantly higher than the expected by chance.
This is now mentioned Lines 431-434.

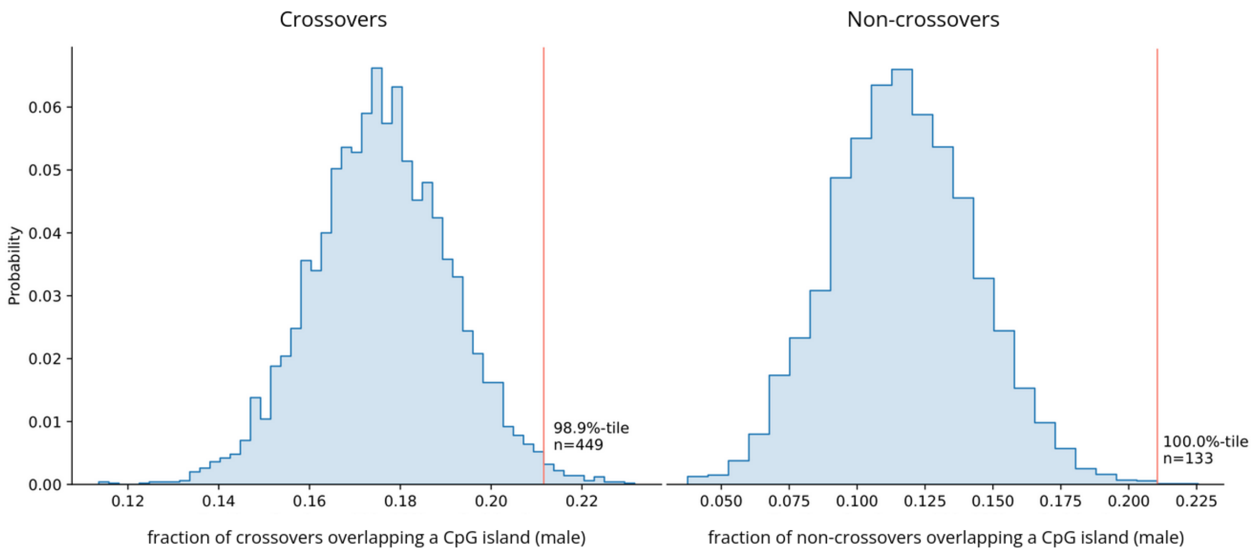

Figure R3. Overlap of both types of recombination events (in males) with CpG islands. Fractions of crossovers (top) and non-crossovers (bottom) identified in males meioses within 100 bps of a CpG island. The vertical lines show the observed overlap. The distribution for the overlap expected by chance is shown as a histogram, obtained by randomly shuffling all the events within a 2.5 Mb window on each side of their original location, matching for the GC content and ensuring similar mappability (see Methods for details).

The Discussion was rather underdeveloped. I agree that there was a lot of direct interpretation in the Results (which was appropriate and helpful), but there are a few open questions I had that could be picked up or discussed, either here or elsewhere:

* More detail on the stabilising selection idea I commented on above.
* The analysis is only going to pick up the "surviving mutations"? I guess that this is probably mitigated to some degree by using embryos, but it could be appropriate to acknowledge that a pedigree-based approach will have some biases.
* What we might miss from the lack of "informative" parts of the genome e.g. only 50% could be examined for mutations.
* DNMs are only informative if both parents homozygous, so how many mutations could potentially be missed due to a lack of information e.g. both parents are heterozygous?

We have now added a paragraph about the conservation of mutation and recombination parameters in the Discussion.

Regarding the other points raised by the reviewer:

While it is true that only mutations compatible with survival in utero will be detected, we expect that to be a rounding error in our estimate of the de novo mutation rate. In humans, for instance, in which each offspring inherits 70 mutations, and fewer than 0.5% of these are strongly deleterious, let alone lethal (Agarwal & Przeworski, 2021). While this estimate is for humans, not birds, it reflects the fact that the mutational target size for lethal mutations is orders of magnitude smaller than the number of base pairs, so the vast majority of DNMs do not affect survival.

As we detail in point 4. raised by reviewer 2, our estimate of mutation rates should be generalizable to most of the genome, but that assumption remains to be verified. We now state this explicitly on line 749-754.

Regarding the last point raised, the denominator for the mutation rate includes all callable sites, including sites at which one or both parents are heterozygous. Such sites represent 1.5% of the total callable sites (which is as expected, given that heterozygosity is ~1%). Therefore they are unlikely to affect our mutation rate estimate. We have added a sentence to clarify this point in the Methods (lines 692-696).

* Small Errors/Suggestions :

Page 15: 36 meiosis -> 36 meioses

Thank you, we modified the text accordingly.

Page 34: "Ancestral alleles and mutation transition matrix" - should this go earlier e.g., as second paragraph of Page 32?

This section is part of the pipeline used to infer a genetic map using the program LDhelmet, so we believe that it should stay where it is currently.

Page 35: define "p values"

We have now done so.

It might be helpful for some readers to define "proband".

We have now done so.

In Figure 1, some females seem to have mated with females!

We thank the reviewer for catching this embarrassing error, which we have obviously fixed.

Throughout the manuscript: Please check that all software versions are provided (COSMIC, VCFtools, etc.)

We have done so.

<u>References</u>:

Agarwal, I., & Przeworski, M. (2021). Mutation saturation for fitness effects at human CpG sites. Elife, 10, e71513.
Bascón-Cardozo, K., Bours, A., Manthey, G., Durieux, G., Dutheil, J. Y., Pruisscher, P., ... & Liedvogel, M. (2024). Fine-Scale Map Reveals Highly Variable Recombination Rates Associated with Genomic Features in the Eurasian Blackcap. Genome Biology and Evolution, 16(1), evad233.
Charmouh, A. P., Sørud, P. P., Hansen, L. T., Besenbacher, S., Winge, S. B., Almstrup, K., ... & Schierup, M. H. (2024). Estimating gene conversion tract length and rate from PacBio HiFi data. bioRxiv, 2024-07.
Cole, F., Keeney, S., & Jasin, M. (2010). Comprehensive, fine-scale dissection of homologous recombination outcomes at a hot spot in mouse meiosis. Molecular cell, 39(5), 700-710.
Halldorsson, B. V., Hardarson, M. T., Kehr, B., Styrkarsdottir, U., Gylfason, A., Thorleifsson, G., ... & Stefansson, K. (2016). The rate of meiotic gene conversion varies by sex and age. Nature genetics, 48(11), 1377-1384.
Harland C, Charlier C, Karim L, Cambisano N, Deckers M, Mni M, Mullaart E, Coppieters W, Georges M. 2017. Frequency of mosaicism points towards mutation-prone early cleavage cell divisions in cattle. : 079863.
Holloway, J. K., Booth, J., Edelmann, W., McGowan, C. H., & Cohen, P. E. (2008). MUS81 generates a subset of MLH1-MLH3–independent crossovers in mammalian meiosis. PLoS genetics, 4(9), e1000186.
Huang, Z., Xu, Z., Bai, H., Huang, Y., Kang, N., Ding, X., ... & Xu, L. (2023). Evolutionary analysis of a complete chicken genome. Proceedings of the National Academy of Sciences, 120(8), e2216641120.
Jónsson H, Sulem P, Kehr B, Kristmundsdottir S, Zink F, Hjartarson E, Hardarson MT, Hjorleifsson KE, Eggertsson HP, Gudjonsson SA, et al.2017. Parental influence on human germline de novo mutations in 1,548 trios from Iceland. Nature 549: 519–522.
Kim, J., Lee, C., Ko, B. J., Yoo, D. A., Won, S., Phillippy, A. M., ... & Jarvis, E. D. (2022). False gene and chromosome losses in genome assemblies caused by GC content variation and repeats. Genome biology, 23(1), 204.
Li, R., Bitoun, E., Altemose, N., Davies, R. W., Davies, B., & Myers, S. R. (2019). A high-resolution map of non-crossover events reveals impacts of genetic diversity on mammalian meiotic recombination. Nature communications, 10(1), 3900.
Liang, X., Yang, S., Wang, D., & Knief, U. (2024). Characterization and distribution of de novo mutations in the zebra finch. Communications Biology, 7(1), 1243.
Lindsay, S. J., Rahbari, R., Kaplanis, J., Keane, T., & Hurles, M. E. (2019). Similarities and differences in patterns of germline mutation between mice and humans. Nature communications, 10(1), 4053.
McAuley, J. B., Servin, B., Burnett, H. A., Brekke, C., Peters, L., Hagen, I. J., ... & Johnston, S. E. (2024). The genetic architecture of recombination rates is polygenic and differs between the sexes in wild house sparrows (Passer domesticus). Molecular biology and evolution, 41(9), msae179.

Porubsky, D., Dashnow, H., Sasani, T. A., Logsdon, G. A., Hallast, P., Noyes, M. D., ... & Eichler, E. E. (2024). A familial, telomere-to-telomere reference for human de novo mutation and recombination from a four-generation pedigree. bioRxiv.
Robledo-Ruiz, D. A., Gan, H. M., Kaur, P., Dudchenko, O., Weisz, D., Khan, R., ... & Pavlova, A. (2022). Chromosome-length genome assembly and linkage map of a critically endangered Australian bird: the helmeted honeyeater. GigaScience, 11, giac025.
Stapley, J., Feulner, P. G., Johnston, S. E., Santure, A. W., & Smadja, C. M. (2017). Variation in recombination frequency and distribution across eukaryotes: patterns and processes. Philosophical Transactions of the Royal Society B: Biological Sciences, 372(1736), 20160455.
Tan, H. Z., Scherer, P., Stuart, K. C., Bailey, S., Lee, K. D., Brekke, P., ... & Santure, A. W. (2024). A high-density linkage map reveals broad-and fine-scale sex differences in recombination in the hihi (stitchbird; Notiomystis cincta). Heredity, 133(4), 262-275.
Wall, J. D., Robinson, J. A., & Cox, L. A. (2022). High-resolution estimates of crossover and noncrossover recombination from a captive baboon colony. Genome biology and evolution, 14(4), evac040.
Williams, A. L., Genovese, G., Dyer, T., Altemose, N., Truax, K., Jun, G., ... & Przeworski, M. (2015). Non-crossover gene conversions show strong GC bias and unexpected clustering in humans. Elife, 4, e04637.
Zhang, H., Lundberg, M., Ponnikas, S., Hasselquist, D., & Hansson, B. (2024). Male-biased recombination at chromosome ends in a songbird revealed by precisely mapping crossover positions. G3: Genes, Genomes, Genetics, 14(9), jkae150.
