# Supplementary material for: Germline mutation rates and fine-scale recombination parameters in zebra finch
Source: PLoS Genet. 2025 Apr 15;21(4):e1011661. doi: 10.1371/journal.pgen.1011661 (PMC12047795; doi:10.1371/journal.pgen.1011661)
Supplement: Attachment — Submitted filename: Response to Reviewers.pdf [file pgen.1011661.s038.pdf]

Reviewer #1: Thank you to the authors for addressing my previous comments. I still feel positive towards the paper and find that it is improved/clearer. It's an impressive amount of robust analysis. However, I still have some comments about the framing and conclusions of the study.

After reading and considering the comments of the other reviewers, I still think that this is not a comparative study (as stated on Line 29) and that the strong emphasis of comparing to mammals (as is done in the title, and for each statement in Lines 34 – 40) is not necessarily justified. Perhaps the authors could argue that comparisons can be invoked in terms of measures that are rare (i.e., de novo mutation). However, in terms of recombination, I think that there have been enough measures in birds and mammals over many years to show that (a) zebra finch recombination rate is probably lower than other passerine species and (b) not all mammals are hovering around 1 cM/Mb, as is clearly stated in lines 68-69 (indeed, this does not match the statement in Lines 61 & 62). The authors have acknowledged this in their response, and extensive discussions in recombination mechanisms (lines 84 – 102 – of which the authors have made a huge contribution) yet strong statements remain. Generally, I agree that comparison is important, but the *strong* emphasis on comparison with mammals detracts from interesting conclusions of this paper that stand on their own merit.

We have thoroughly revised the Abstract and Introduction to tone down the emphasis on the comparison to mammals, as well as made changes to the Discussion. We have also changed the title accordingly.

To clarify, we agree that there have been many studies of recombination in birds and mammals. Few, however, focused on fine-scale properties of recombination resolutions: to our knowledge, there is one other study in birds to identify non-crossover events or estimate biased gene conversion, and none that estimates tract lengths or the ratio of crossover to non-crossovers. Even in mammals, only a handful of species have been characterized in this regard. Thus, the novelty of our study lies in the findings about these fine-scale properties of recombination, which appear to be similar to what has been reported in mammals.

With regard to mutation, our estimate of the mutation rate is the most precise to date for birds, being based on more meioses, and it is very similar to those of mammals with similar generation times (on the order of years) (see Figure S20). More generally, the mutation rates per generation in birds and mammals studied to date appear to follow a very similar relationship to generation time (Figure S20).

Similarly, I am still not convinced that the current manuscript offers any novel conclusions on natural selection on recombination. The authors invoke arguments related to genetic variation persisting in populations (Lines 597 – 600), but don't spell out why this is relevant. The authors have also shown throughout the manuscript that recombination is highly variable across birds and mammals. I don't disagree that there is likely selection occurring (indeed, selective constraints have been discussed over decades of literature summarised in various reviews, e.g. Arter & Keeney 2024, Stapley et al 2017,

Bomblies & Henderson 2022, etc. etc.), but I think the authors oversell the contribution of this manuscript in answering this question.

What we meant to say is that the presence of modifier alleles that influence mutation and recombination properties, as well as other lines of evidence (e.g., heritability estimates), establish that these properties are partly under genetic control and therefore could evolve. That many properties of recombination resolutions remain so similar nonetheless over hundreds of millions of years hints at stabilizing selection, as noted by reviewer 2. We have revised the Discussion to hopefully clarify this point, and tone down our previous claims.

We respectfully disagree, however, that we have shown "throughout the manuscript that recombination is highly variable across birds and mammals". Instead our results show that fine-scale properties of recombination resolutions, including the degree of biased gene conversion, length of the conversion tract, and ratio of non-crossover to crossover are similar to estimates for mice and primates. We have now added fine-scale to the title and revised our Introduction to emphasize that our focus is on these aspects, rather than broad scale properties. We agree that this work does not answer the question of how selection acts on recombination, or even begin to answer it; it only adds another data point over a broader taxonomic distance. In fact, we explicitly state (as we had in the previous version) that we were only raising the possibility and that "The increasing availability of genome sequences should make it possible to test this hypothesis more systematically, and to identify which aspects of mutagenesis and meiotic recombination are most strongly conserved."

In this regard, we believe that we are making a distinct point than the references provided by the reviewers, as Stapley et al. and Bomblies and Henderson focus on variation on recombination on broad-scales and its adaptive explanations, rather than conservation of fine-scale recombination properties. In turn, Arter & Keeney focus on a related but distinct point, namely why recombination proteins evolve so rapidly despite the importance and conservation of recombination processes. We now cite all three references in our final paragraph.

Line 33: information "for or from" 80 meioses?

Changed accordingly

Lines 74-75: The Arter and Keeney review would support conservation of mechanisms over broad phylogenetic distances.

We thoroughly modified the structure of the introduction and no longer make this point, so we did not add this reference here, but instead on line 611.

Lines 128 – 130: At this point of reading, I don't know what the COSMIC mutational signatures are.

We have changed the wording in the Introduction, and introduce the COSMIC signatures when we use them.

Lines 589 – 590: But the Priore and Pigozzi paper shows remarkable variation in CO count using MLH1 foci.

We had considered that a 2-fold difference in mean recombination rate was relatively small, given the phylogenetic distance, but we agree that the emphasis of the paper is on the differences between guinea fowl and chicken, so have removed this reference. We instead reference it when discussing the distribution of crossovers that we find on macro-chromosomes, which echoes what they reported in guinea fowl.

Reviewer #2: The manuscript represents a comprehensive analysis of the characteristics of mutation and meiotic recombination in zebra finch. I still think that this study provides important and valuable data and is useful for researchers in the fields of avian genomics and evolution. For the present version, authors carefully addressed the comments and performed additional analyses. Please find below my comments on the current version of the manuscript.

1) For the comment about stabilizing selection on recombination characteristics, I understand the authors' arguments regarding their similarity between taxa despite millions of years of evolution. However, as the authors rightly point out, discussing the scale of RR variation between taxa is beyond the scope of this study. As well as inferring selection regimes of recombination modifiers. I think the current version (with slightly modified title, abstract and toned-down claims) better aligns with the study's aims.

We thank the reviewer for these comments, and have added the word "fine-scale" to the title to be more precise about our claims (and revised title, abstract and Introduction).

2) Following the comment about double crossovers and the potential detection of a type II non-interfering pathway of CO resolution in birds, authors analyzed their data and claimed that 8% of all 1,174 detected crossovers are located closer than 1 Mb to their neighbor. The authors also suggest that these instances may have resulted from complex resolutions with template switching.

I was interested in this data and decided to take a closer look at it.

From S3 Table, there are 126 chromatids with more than one CO (293 COs in total). Among them, 38 chromatids (30%) contain COs located closer to each other than 1 Mb (with an average distance of 277 Kb). Interestingly, they are distributed very unevenly throughout the genome. Of these 38 chromatids, 17 (45%) belong to microchromosomes from 25 to 37. If we include COs separated by a distance of 1 to 2 Mb, this percentage increases to 50%. One theoretical possibility that comes to mind is that the type II pathway may act predominantly in microchromosomes as a backup mechanism to ensure their correct segregation. However, the complete absence of such events on most medium-sized chromosomes

(14-15, 17-24) seems strange.

Moreover, among the 126 chromatids, there were 20 with more than two COs located relatively close to each other (74 COs in total), with 6 of them including "complex" COs. For example, there are four cases on chromosome 3, where 3 or 5 COs are located within the distance of ~650 kb. Moreover, all four cases are located in the same chromosome region. There is also a similar region on chromosome 2 (where 3 cases of 3 COs are located within the same ~800 kb region). The same applies to 6 cases on chromosome 13, which are slightly more widely distributed but also include up to 5 crossovers.

While it is theoretically possible that high-intensity hotspot usage may be responsible for the repeated inclusion of a region in recombination, it does not explain multiple template switching. The type II pathway also does not convincingly explain this (and it is also questionable whether it plays a role, given the skewed distribution of "non-interfering" COs across the genome). Does the phenomenon of complex resolutions with template switching sufficiently explain such instansies?—How long and complex could these events be?

While hypothesising on these questions might be out of the scope of this paper, it is necessary to ensure that these data are not caused by some kind of technical and/or methodological issue and to determine whether it is reliable to consider such "complex-complex" events as 3/5 COs instead of a single event.

We agree, and thank the reviewer for the original nudge and for conducting this analysis. Following their lead, we investigated genomic regions with a suspiciously high density of crossover events within the same meiosis. We clustered events by grouping crossovers occurring within 2 Mb of each other and identified 17 clusters containing three or more events, totaling 55 crossovers. As the reviewer noted, these clusters happened to be in the same regions across different meiosis; specifically, we identified nine such regions spanning a total of 6 Mb. Given that these instances are likely spurious, we removed the crossovers within these regions (86 events in total), resulting in a revised dataset of 1,088 crossovers. We then re-ran all the analysis with this updated dataset. Other than the percent of crossovers that appear to be class II (now ~3%), none of the results changed qualitatively; indeed, the results remain very similar quantitatively as well. The additional filtering steps are described in the Methods, and the numbers were updated throughout the manuscript. We are grateful to them for spotting this issue.

3) Regarding the minor comment about references (6) Please revise the reference list. For example, the citation of Soler et al. (2021) from p.4 is missing), the authors stated that this issue was fixed, but the current reference does not appear to match the described content—"primordial germ cell phenotypes before gonadal development" (line 83). Could you please clarify?

The reference should be:
Soler L, Alves S, Brionne A, Jacques A, Guérin V, Cherif-Feildel M, Combes-Soia L, Fouchécourt S, Thélie A, Blesbois E, *et al.*2021. Protein expression reveals a molecular sexual identity of avian primordial germ cells at pre-gonadal stages. *Scientific Reports* 11: 19236.

We apologize for this mistake; we have now double-checked the references and there are hopefully no more such errors.

I also have a couple of other comments.

4) line 538: the observed number of crossovers used to infer the NCO:CO ratio (Table 1) is 18.5. I assume this number results from removing nine smallest microchromosomes from the crossover dataset (as was done for non-crossovers) but I didn't find any mention of this in either Results or Methods sections (which I think would be good to have).

Indeed; we have now added this information.

5) in the table S4, the number of crossover events and the p-value (the second column) do not correspond to the data in lines 289-292.

We apologize; we have now updated the numbers in Table S4.

Reviewer #3: The authors have satisfactorily addressed my initial comments and have also included new analyses suggested by the other referee.
